# Engineering of anticancer human immunoglobulin A equipped with albumin for enhanced plasma half-life

**DOI:** 10.1093/pnasnexus/pgaf042

**Published:** 2025-02-11

**Authors:** Simone Mester, Chilam Chan, Marta Lustig, Stian Foss, J H Marco Jansen, Marie Leangen Herigstad, Mitchell Evers, Jeannette Nilsen, Karli R Reiding, J Mirjam A. Damen, Renate Burger, Algirdas Grevys, Bjørn Dalhus, Thomas Valerius, Inger Sandlie, Jeanette H W Leusen, Jan Terje Andersen

**Affiliations:** Department of Immunology, Oslo University Hospital Rikshospitalet and University of Oslo, Norway, PO Box 4950, N-0424 Oslo, Norway; Precision Immunotherapy Alliance (PRIMA), Department of Pharmacology, Institute of Clinical Medicine, University of Oslo, PO Box 1171, N-0318 Oslo, Norway; Center for Translational Immunology, University Medical Centre Utrecht, 3584CX Utrecht, The Netherlands; Division of Stem Cell Transplantation and Immunotherapy, Department of Medicine II, Christian Albrechts University Kiel and University Medical Center Schleswig-Holstein, 24105 Kiel, Germany; Department of Immunology, Oslo University Hospital Rikshospitalet and University of Oslo, Norway, PO Box 4950, N-0424 Oslo, Norway; Precision Immunotherapy Alliance (PRIMA), Department of Pharmacology, Institute of Clinical Medicine, University of Oslo, PO Box 1171, N-0318 Oslo, Norway; Center for Translational Immunology, University Medical Centre Utrecht, 3584CX Utrecht, The Netherlands; Department of Immunology, Oslo University Hospital Rikshospitalet and University of Oslo, Norway, PO Box 4950, N-0424 Oslo, Norway; Precision Immunotherapy Alliance (PRIMA), Department of Pharmacology, Institute of Clinical Medicine, University of Oslo, PO Box 1171, N-0318 Oslo, Norway; Center for Translational Immunology, University Medical Centre Utrecht, 3584CX Utrecht, The Netherlands; Department of Immunology, Oslo University Hospital Rikshospitalet and University of Oslo, Norway, PO Box 4950, N-0424 Oslo, Norway; Precision Immunotherapy Alliance (PRIMA), Department of Pharmacology, Institute of Clinical Medicine, University of Oslo, PO Box 1171, N-0318 Oslo, Norway; Biomolecular Mass Spectrometry and Proteomics, Bijvoet Center for Biomolecular Research and Utrecht Institute for Pharmaceutical Sciences, University of Utrecht, and Netherlands Proteomics Center, 3584 CH, Utrecht, The Netherlands; Biomolecular Mass Spectrometry and Proteomics, Bijvoet Center for Biomolecular Research and Utrecht Institute for Pharmaceutical Sciences, University of Utrecht, and Netherlands Proteomics Center, 3584 CH, Utrecht, The Netherlands; Division of Stem Cell Transplantation and Immunotherapy, Department of Medicine II, Christian Albrechts University Kiel and University Medical Center Schleswig-Holstein, 24105 Kiel, Germany; Department of Immunology, Oslo University Hospital Rikshospitalet and University of Oslo, Norway, PO Box 4950, N-0424 Oslo, Norway; Precision Immunotherapy Alliance (PRIMA), Department of Pharmacology, Institute of Clinical Medicine, University of Oslo, PO Box 1171, N-0318 Oslo, Norway; Department of Medical Biochemistry, Institute of Clinical Medicine, University of Oslo, PO Box 1171, N-0318 Oslo, Norway; Department for Microbiology, Oslo University Hospital, Rikshospitalet, PO Box 4950, N-0424 Oslo, Norway; Division of Stem Cell Transplantation and Immunotherapy, Department of Medicine II, Christian Albrechts University Kiel and University Medical Center Schleswig-Holstein, 24105 Kiel, Germany; Department of Immunology, Oslo University Hospital Rikshospitalet and University of Oslo, Norway, PO Box 4950, N-0424 Oslo, Norway; Department of Biosciences, University of Oslo, N-0316 Oslo, Norway; Center for Translational Immunology, University Medical Centre Utrecht, 3584CX Utrecht, The Netherlands; Department of Immunology, Oslo University Hospital Rikshospitalet and University of Oslo, Norway, PO Box 4950, N-0424 Oslo, Norway; Precision Immunotherapy Alliance (PRIMA), Department of Pharmacology, Institute of Clinical Medicine, University of Oslo, PO Box 1171, N-0318 Oslo, Norway

**Keywords:** IgA, albumin, FcRn, engineering, half-life, Biological Sciences, Immunology and inflammation

## Abstract

Most therapeutic antibodies are based on immunoglobulin G (IgG) due to their potent effector functions and long plasma half-life. However, also monomeric IgA has emerged as an attractive candidate for cancer treatment as, upon specific binding to tumor cells, it can activate myeloid cells, like polymorphonuclear leukocytes and macrophages, to kill the tumor cells by engaging the Fc α receptor I (FcαRI). Despite this favorable property, human IgA has a short plasma half-life in both mice and humans, which is clearly limiting preclinical studies in a translational perspective. Here, we report on albumin-equipped designs of human IgA antibodies that are long acting due to tailored binding to the human form of neonatal Fc receptor (FcRn), which is a natural plasma half-life regulator of albumin. Importantly, this was achieved without compromising the ability of IgA to engage and activate FcαRI-expressing effector cells for tumor cell killing in vitro and in vivo in a new mouse model transgenic for the human forms of FcRn and FcαRI. We further show that the potency of the engineered long-acting human IgA against tumor cells with intermediate target antigen expression levels could be enhanced by myeloid checkpoint inhibitors targeting the signal regulatory protein α-CD47 axis.

**Significance Statement**

The clinical success of therapeutic monoclonal immunoglobulin G (IgG) antibodies has spurred an intense interest in exploring new formats, including the use of other antibody isotypes than IgG. In the context of cancer, monomeric human IgA has shown promising preclinical results, as it can mediate potent cancer cell killing via engagement of the Fc α receptor I (FcαRI), expressed on polymorphonuclear leukocytes, such as neutrophils. However, a major drawback in a translational perspective is its short plasma half-life. By leveraging human neonatal Fc receptor (FcRn) biology, we have engineered IgA formats fused with albumin, which engage both FcRn and FcαRI favorably, resulting in extended plasma half-life and eradication of cancer cells.

## Introduction

More than 100 monoclonal immunoglobulin G (IgG) antibodies have been approved for clinical use, of which the majority are tailored for treatment of cancer (1). One reason for this success is that such IgG antibodies could mediate killing of the targeted cancer cells via engagement of the complement cascade or Fc γ receptors (FcγRs) expressed by distinct immune effector cells (2, 3). Another unique feature of IgG is that it naturally has a plasma half-life of 3 weeks on average in humans (4, 5), which guides dosing and frequency of administration and impacts patient compliance. However, while monoclonal IgG-based therapy has a major impact on the treatment of several cancer types, long-term clinical efficacy or cure of disease is often not achieved. Consequently, there is a need for antibody designs with a variety of potent modes of action (2, 6).

Interestingly, it was recently shown that successful immunotherapies expand the neutrophil population in the tumor (7), while another study demonstrates that engineered T cells directed against tumor antigens can activate otherwise suppressive neutrophils to attack the tumor, even after T-cell therapy escape (8). As such, strategies that can further strengthen the potential of neutrophils to eradicate tumor cells in concert with other immunotherapies should be highly attractive to explore. In this regard, one attractive strategy would be to take advantage of the IgA class of antibodies. The reason for this is that monomeric human IgA can cross-link Fc α receptor I (FcαRI) expressed on polymorphonuclear (PMN) leukocytes, such as neutrophils, and induce potent reactive oxygen species production, phagocytosis, neutrophil extracellular trap formation, cytokine release, as well as antibody-dependent cellular cytotoxicity (ADCC) directed against the tumor cells, as reviewed (9, 10).

Importantly, IgA is significantly more efficient in inducing these effector functions of PMNs than IgG (11–16). In blood, PMNs are the most abundant leukocytes, and they constitutively express FcαRI. The profound ability of human IgA to activate PMNs has been demonstrated for tumor-relevant antigens, such as EpCAM (14), HLA class II (17), carcinoembryonic antigen (18), CD20 (15), epidermal growth factor receptor (EGFR) (12), and human EGFR 2 (HER2) (19), CD38 (20), and GD2 (21, 22). It has also been shown that the anticancer effect of IgA can be enhanced when combined with checkpoint inhibitors targeting the signal regulatory protein α (SIRPα)-CD47 axis (23). Despite these encouraging preclinical data, the immunotherapeutic potential of IgA has not yet been explored in humans, although engineering of human IgA is being investigated (9). One major obstacle in this regard is its limited plasma half-life of no more than 1 day in mice and 5 days in humans (12, 19, 24, 25), which hampers preclinical testing in mouse models as well as clinical translation, where multiple and frequent injections of large quantities of IgA would be required to reach effective therapeutic concentrations.

IgA is produced by B cells and plasma cells in about twice the amounts than IgG, but the concentration of IgA in blood is five times lower than that of IgG (∼2 and 10 mg/mL, respectively), which is explained by the more rapid elimination of IgA from the circulatory system (26). In contrast, the long plasma half-life of IgG is secured by a neonatal Fc receptor (FcRn)-dependent mechanism, where IgG is rescued from intracellular degradation (27, 28). FcRn is predominantly expressed within acidified endosomes in a range of cell types and binds IgG taken up by fluid-phase pinocytosis followed by recycling to the cell surface, where IgG is released upon exposure to the neutral pH of blood. Importantly, albumin also binds FcRn (29–31) and is rescued by a similar pH-dependent recycling mechanism (32, 33), although the receptor binding sites of the two are distinct and nonoverlapping (29, 30, 34).

Thus, by taking advantage of human FcRn biology and its relationship with its ligands, we report here on molecular designs where we have fused human IgA with engineered human serum albumin (HSA), which resulted in favorable FcRn engagement and rescue from intracellular degradation. This further translated into an extended plasma half-life of tailored IgA designs in a transgenic mouse model expressing human FcRn. Importantly, we demonstrate that the IgA designs could cross-bind FcαRI on human PMNs and induce potent anticancer cell activity both in vitro and in vivo. Anticancer activity could also be further enhanced by myeloid checkpoint inhibition targeting CD47. As such, combining albumin-based domains with IgA was shown to be a feasible strategy to overcome the limiting short plasma half-life of anticancer IgA1 and IgA2 antibodies.

## Results

### AlbuAb: human IgA equipped with albumin

**Fig. 1.**
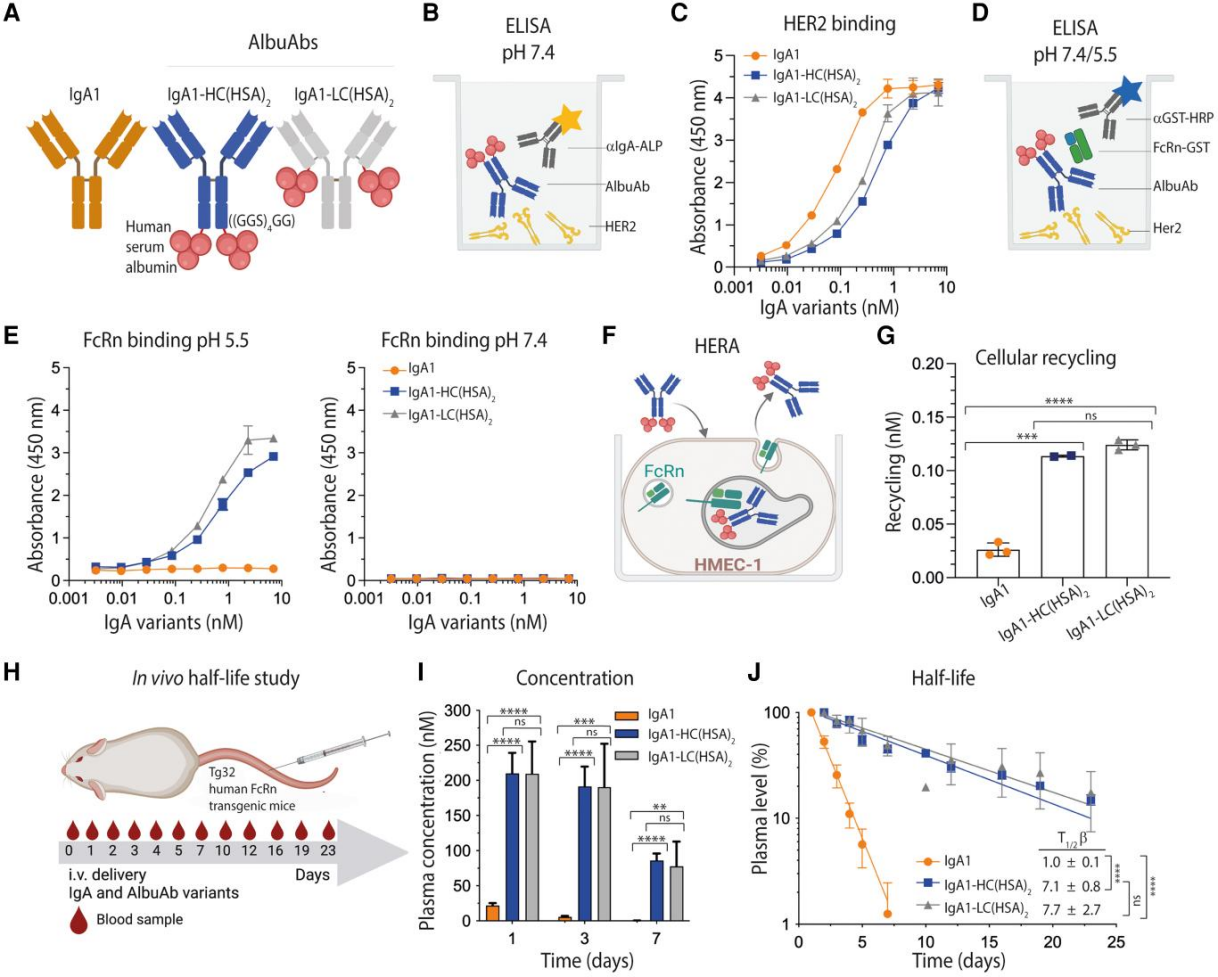
Fusion of full-length HSA to IgA1 extends its plasma half-life in human FcRn-expressing mice. A) Schematic illustration of IgA1 and HSA genetically fused to either the HCs or LCs of human IgA1 via a flexible glycine–serine linker ((GGS)_4_GG). The tailpiece of the IgA1 HCs was deleted. B) Schematic illustration of the HER2-binding ELISA setup. C) ELISA results showing binding of titrated amounts of IgA1 and IgA1-HSA fusions to recombinant HER2 coated in wells at pH 7.4. Shown as mean ± SD of duplicates. D) The setup for FcRn-binding ELISA. E) ELISA showing binding of titrated amounts of IgA1 and IgA1-HSA fusions to recombinant GST-tagged human FcRn at pH 5.5 and 7.4. Shown as mean ± SD of duplicates. F) Overview of HERA. G) 400 nM of IgA1 and IgA1-HSA fusions were added for 4 h to human FcRn-expressing HMEC-1 cells, followed by extensive washing and incubation overnight. The amounts of the antibody variants present in collected medium samples were quantified by ELISA. Shown as mean ± SD of triplicates from one representative biological replicate. ns > 0.05, ***P = 0.0003, ****P < 0.0001, by using unpaired t test. H) Illustration of the in vivo half-life study setup. I) Plasma concentration (nM) at days 1, 3, and 7 of IgA1 and IgA1-HSA fusions. J) Log-linear changes in the plasma level (%) of IgA1 and IgA1-HSA fusions in human FcRn transgenic mice. The IgA1 variants were administrated as a single i.v. injection to five mice per group. The data are represented as mean ±SD. ns > 0.05, ***P = 0.0002, **P = 0.0012, ****P < 0.0001, by two-tailed analysis using unpaired t test. Schematic figures were made with BioRender.

Albumin is a nonglycosylated, effector negative molecule with a favorably long plasma half-life in humans, which makes it an attractive fusion partner for half-life extension (35). As such, we tailor-designed constructs where full-length HSA was genetically fused to the C-terminal end of either both heavy chains (HCs) or both light chains (LCs) of human IgA1 via a flexible glycine–serine linker ((GGS)_4_GG; Fig. 1A). To exclude the formation of dimers of such AlbuAb constructs, the tailpiece at the C-terminal HC end, containing free cysteine residues, was deleted. The fusions were made with specificity for HER2 using the variable domains derived from the monoclonal IgG1 antibody trastuzumab. The expression vectors were transiently transfected into human embryonic kidney (HEK) 293E cells, followed by the collection of supernatants, purification of the secreted proteins on an antihuman IgA CaptureSelect column, and then size exclusion chromatography (SEC). This yielded pure fractions as shown by analytical SEC (Fig. S1).

### HSA-fused IgA1 binds cognate antigen

To address whether the presence of fused HSA affected the binding of IgA1 to cognate antigen, titrated amounts of the fusions and unfused IgA1 were compared for binding to recombinant human HER2 coated in an enzyme-linked immunosorbent assay (ELISA; Fig. 1B). This revealed that the HSA-fused IgA variants bound HER2 but with reduced activity when fused to the HCs and the LCs compared with unfused IgA1 (Fig. 1C and Table S1). The same HER2-binding phenotype was shown at different pH conditions, ranging from pH 7.4 to 6.0 (Fig. S2 and Table S1).

### Human FcRn engagement results in extended plasma half-life

To study whether the fusion of full-length HSA could provide IgA1 with the ability to engage human FcRn, the AlbuAbs were captured on HER2, followed by adding recombinant human FcRn in ELISA (Fig. 1D). The results showed strong binding at acidic pH, while no binding was measured at pH 7.4 (Fig. 1E). To study this in a cellular context, a human endothelial cell–based recycling assay (HERA) (33) was performed to measure the ability of the variants to be rescued from intracellular degradation upon exposure to the FcRn-expressing cells (Fig. 1F). The results revealed that attaching HSA to either the LCs or HCs of IgA1 results in enhanced recycling and, as such, improved rescue from intracellular degradation, compared with naked IgA1 (Fig. 1G).

These encouraging in vitro results motivated us to determine their plasma half-lives in a relevant mouse model. As mouse FcRn binds human albumin poorly, which excludes conventional mice from preclinical evaluation of HSA-based designs (36–42), we took advantage of transgenic mice expressing human FcRn and lacking the murine receptor as well as mouse albumin (39). The AlbuAbs were compared with that of naked human IgA1 following intravenous injections and collection of blood samples for up to 23 days (Fig. 1H). The amounts present at the different time points were quantified in ELISA. The data revealed that the plasma concentrations were drastically higher 1 day post injection, reaching 10-fold higher levels of the HSA-fused IgA1 variants compared with naked IgA1 (Fig. 1I). While unfused IgA1 was rapidly cleared from blood, 30-fold more of the HSA-fused IgA1 variants were detected at day 3 (Fig. 1I). This resulted in half-lives of 7.1 and 7.7 days for the HC- and LC-fused variants, respectively, which were 7- to 8-fold longer than that of the 1-day half-life of naked IgA1 when estimated based on the percent remaining in plasma over time (Fig. 1J). Fitting of the plasma concentrations to a noncompartmental pharmacokinetic (PK) model (43) revealed a >50-fold higher area under the curve (AUC) and slower clearance values for the HSA-fused IgA1 variants (1.5–1.9 mL/day/kg) compared to unfused IgA1 (105.3 mL/day/kg; Table S2). This model calculated a 10- to 11-fold extended plasma half-life for the HSA-fused variants, where HC-fused IgA1 showed a slightly longer half-life (10 days) than the LC-fused variant (9.6 days). Notably, the plasma half-life of the corresponding anti-HER2 IgG1 was measured to be comparable in a previous study, reaching 7.5 days (44).

### HSA-fused IgA1 engages human FcαRI

**Fig. 2.**
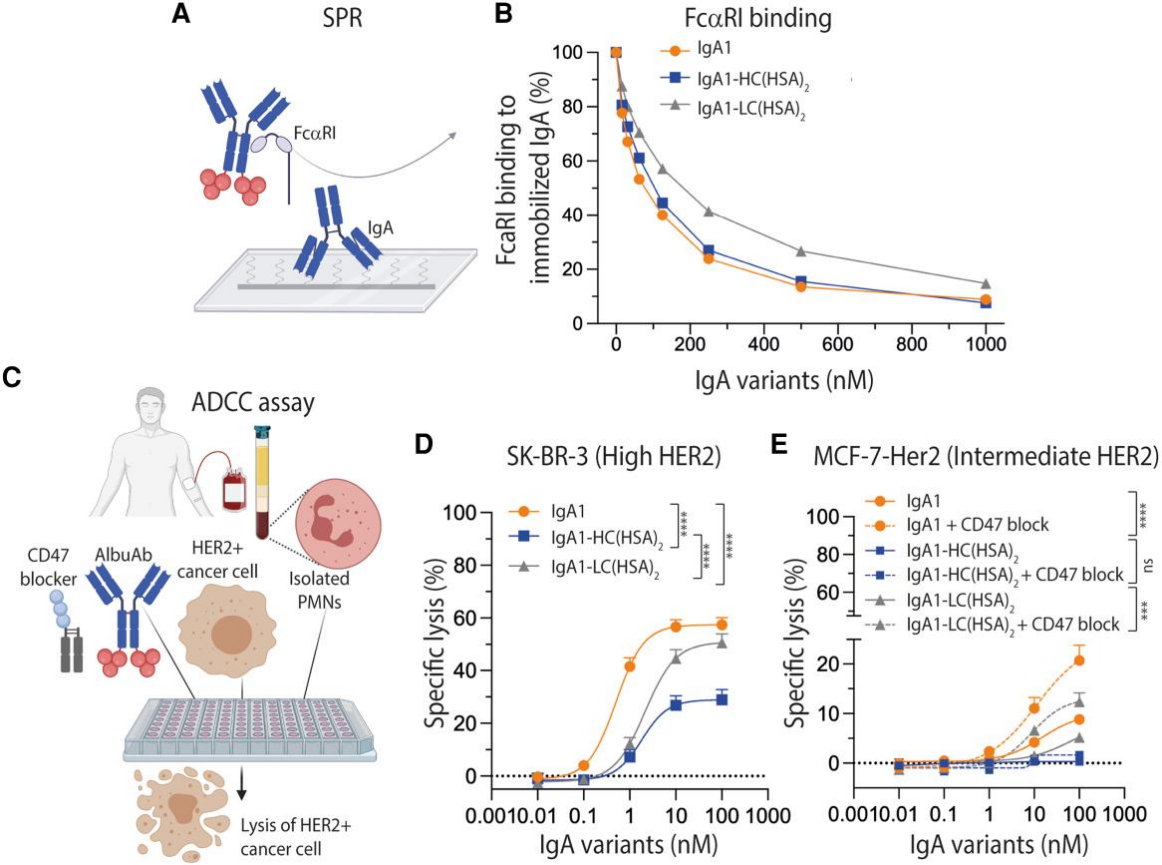
HSA-fused IgA1 engages human FcαRI and induces PMN-mediated ADCC. A) Schematic illustration of the human FcαRI competition SPR setup. B) Competitive binding of a constant amount of His-tagged human FcαRI (100 nM) alone or with titrated amounts of IgA1 and IgA1-HSA (15.6–1,000.0 nM) over immobilized IgA1. C) Schematic illustrations of the ADCC assay. D and E) ADCC assay showing the cytotoxic potential of IgA1 compared with that of the IgA1-HSA fusions in the presence of PMNs (effector cell:target cell [*E*:*T*] = 40:1). E) The cytotoxic potential was studied both in the presence (dotted line) and absence (solid line) of CD47 blockade for all three variants. Shown as mean ± SEM of triplicates from three independent experiments using PMNs from three different donors. ns > 0.05, ***P = 0.0006, ****P < 0.0001, by two-way ANOVA. Specific lysis (%) of (D) SK-BR-3 and (E) MCF-7-HER2 cells was determined. Schematic figures were made with BioRender.

If HSA-fused IgA1 is to be used in a therapeutic setting, binding to FcαRI must be intact. As such, to study the ability of the AlbuAbs to bind to the receptor, we performed a competitive surface plasmon resonance (SPR) assay where titrated amounts of unfused and HSA-fused IgA1 variants were preincubated with recombinant soluble human FcαRI followed by injection over immobilized IgA1 (Fig. 2A). Here, the HC-fused variant was shown to compete for receptor binding almost as efficiently as IgA1, while the LC-fused variant showed reduced capacity (Fig. 2B). Taken together, the fusion of full-length HSA to the LCs of IgA1 reduced FcαRI binding, while fusion to the HC of IgA1 did not.

### AlbuAb targeting of cancer cells mediates killing via FcαRI-expressing neutrophils

To investigate the combined effect of target binding and Fc-mediated FcαRI engagement in a cellular ADCC assay, titrated amounts of the anti-HER2 AlbuAbs were added to freshly isolated human PMNs and HER2-expressing cancer cell lines (Fig. 2C), either expressing high (SK-BR-3, Fig. 2D) or intermediate (MCF-7-HER2, Fig. 2E) HER2 levels on their cell surface (Fig. S3). In both cases, ADCC activity was induced by both formats, but with 1.1- to 2.0-fold reduced capacity of the HSA-fused IgA1 variants compared with naked IgA1 at the highest tested concentration (Fig. 2D and E). However, in contrast to the competition assay, the most prominent reduction in ADCC activity was measured when HSA was fused to the HCs (Fig. 2D and E).

While the half-life extension of the AlbuAbs was superior to that of naked IgA1, their cellular ADCC activity was slightly reduced. We therefore investigated if AlbuAb-mediated ADCC could be enhanced by the combination with blockade of the SIRPα-CD47 axis in the presence of intermediate levels of HER2. As such, the ADCC assay was performed in the presence of CD47 blockade using a truncated human SIRPα fused to an IgG1 Fc harboring effector silencing amino acid substitutions (P329G/L234A/L235A; PGLALA) (45). The results demonstrated enhanced ADCC activity of both unfused IgA1 and HSA-fused IgA1 variants; however, IgA1 with CD47 blockade was superior to the other tested conditions (Fig. 2E).

Taken together, fusing full-length HSA to IgA1 considerably enhanced the availability of IgA in blood and extended its plasma half-life in a relevant preclinical mouse model, taking human FcRn biology into consideration. In addition, combination therapy with CD47 blockade enhanced ADCC-mediated tumor cell killing of IgA1 and the HSA-fused IgA1 variants.

### Albumin-DIII-fused IgA1 is not rescued by FcRn

**Fig. 3.**
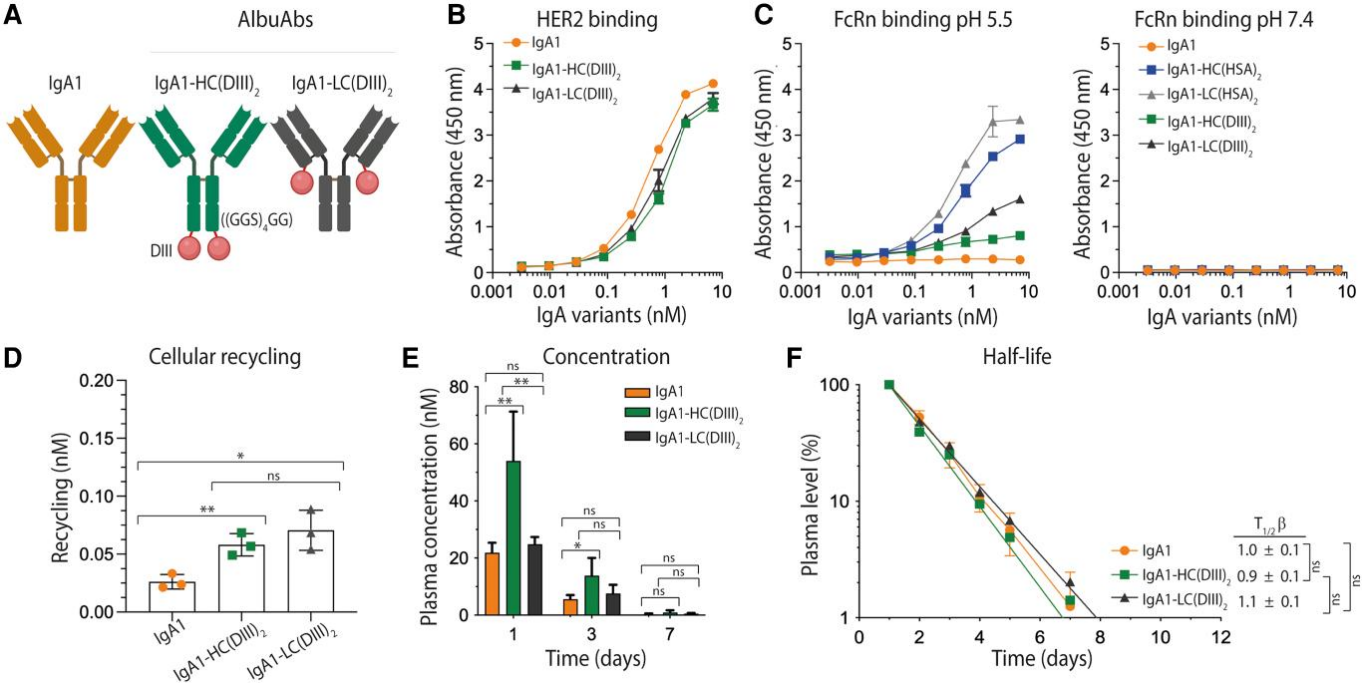
Albumin-DIII-fused IgA1 does not extend plasma half-life. A) Schematic illustration of IgA1 and DIII from HSA genetically fused to either the HCs or LCs of human IgA1 via a flexible glycine–serine linker ((GGS)_4_GG). The tailpiece of the IgA1 variants was deleted. The figure was made with BioRender. B) ELISA results showing binding of titrated amounts of IgA1 and IgA1-DIII fusions to recombinant HER2 coated in wells at pH 7.4. Shown as mean ± SD of duplicates. C) ELISA results showing binding of titrated amounts of IgA1 and IgA1-DIII fusions to recombinant FcRn-GST at pH 5.5 and 7.4. Shown as mean ± SD of duplicates. D) 400 nM of IgA1 and IgA1-DIII variants were added for 4 h to human FcRn-expressing HMEC-1 cells, followed by extensive washing and incubation overnight. The amounts present in collected medium samples were quantified by ELISA. Shown as mean ± SD of triplicates from one representative biological replicate. ns > 0.05, * *P* = 0.014, **P = 0.0086, by using unpaired t test. E) Plasma concentration (nM) at days 1, 3, and 7 of IgA1 and IgA1-DIII fusions. F) Log-linear changes in the plasma level (%) of IgA1 and IgA1-DIII fusions in human FcRn transgenic mice. The IgA1 variants were administrated as a single i.v. injection to five mice per group. The data are represented as mean ± SD. ns > 0.05, **P = 0.0057, 0.0035, *P = 0.0201, by two-tailed analysis using unpaired t test.

Next, we aimed to address if reducing the size of HSA could compensate for the reduced ADCC activity without affecting the ability to engage human FcRn favorably. To test this possibility, we replaced full-length HSA (66.5 kDa) with that of its C-terminal domain, DIII, which is about 23 kDa (Fig. 3A). The rationale is based on the fact that HSA consists of three domains (DI, DII, and DIII), where DIII harbors the principle FcRn-binding site (30, 40, 46). Based on this, we fused wild-type (WT) DIII to the two HCs or LCs, followed by production as above (Fig. S1). While the DIII-fused IgA1 variants bound almost equally well to HER2 as that of IgA1 (Fig. 3B and Table S1), binding to human FcRn (Fig. 1D) was weaker at acidic pH compared to full-length HSA-fused IgA1 (Fig. 3C). Despite this, the DIII-fused IgA1 variants were more efficiently rescued from cellular degradation than unfused IgA1 in HERA (Fig. 3D).

To further study the capacity of DIII to increase exposure and extend the plasma half-life of IgA, the DIII-fused variants were injected in human FcRn transgenic mice, as described above. The measured concentrations in blood were about 10-fold lower than for the HSA-fused IgA variants, with only about 2-fold higher levels of the HC-fused variant at days 1 to 3, while the LC-fused variant was present in similar levels to that of IgA1 (Fig. 3E). Thus, the plasma half-lives of the DIII-fused variants were about 1 day (Fig. 3F), which was also the case using the noncompartmental PK model (Table S2). As such, the strategy failed, despite the fact that we have previously shown that recombinant DIII can engage human FcRn in a pH-dependent manner, however, the lack of in vivo rescue is likely explained by the 10-fold weaker binding affinity of DIII toward the receptor compared with that of full-length HSA (30, 40).

### Engineered DIII-QMP rescues AlbuAbs from intracellular degradation

**Fig. 4.**
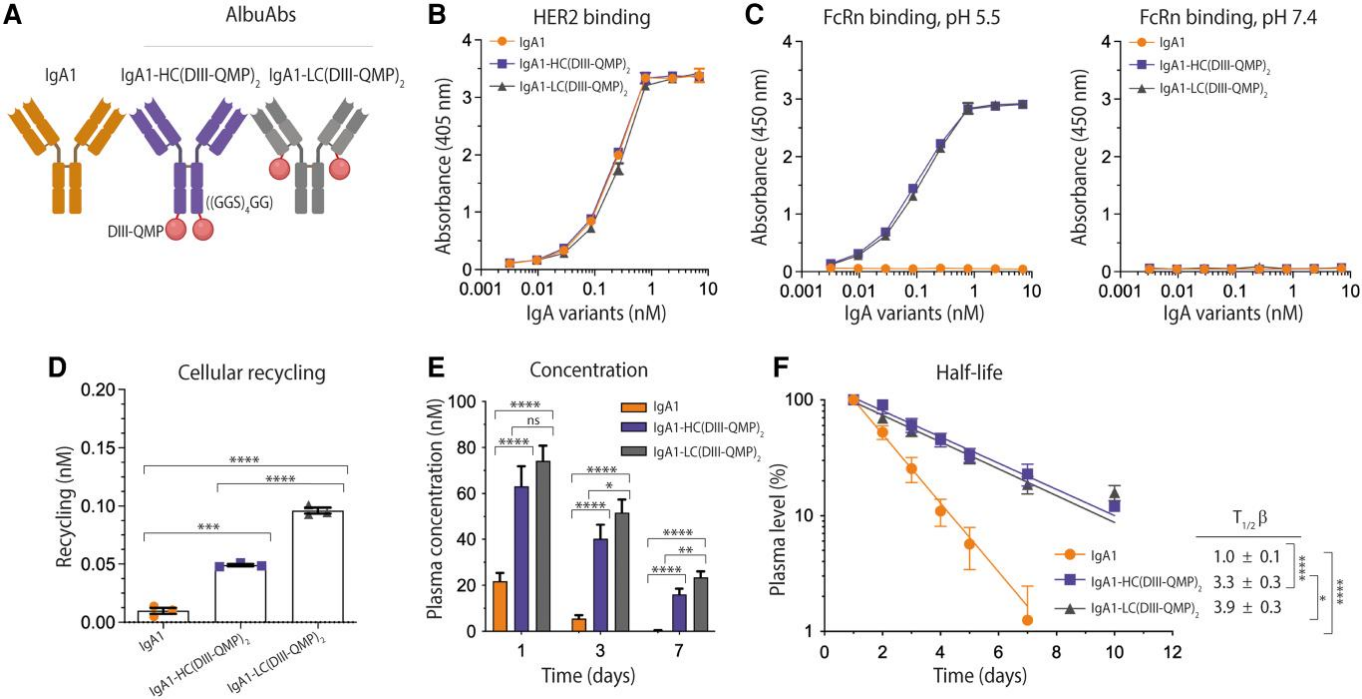
Engineered DIII-QMP extends the plasma half-life of IgA1 via FcRn engagement. A) Schematic illustration of IgA1 and DIII-QMP genetically fused to either the HCs or LCs of human IgA1 via a flexible glycine–serine linker ((GGS)_4_GG). The tailpiece was deleted. The figure was made with BioRender. B) ELISA results showing binding of titrated amounts of IgA1 and IgA1-DIII-QMP fusions to recombinant HER2 coated in wells at pH 7.4. Shown as mean± SD of duplicates. C) ELISA results showing binding of GST-tagged human FcRn at pH 5.5 and pH 7.4 to titrated amounts of IgA1 and DIII-QMP-fused IgA1 variants captured on HER2 coated in wells. Shown as mean ± SD of duplicates. D) 400 nM of IgA1 and IgA1-DIII-QMP variants was added for 4 h to human FcRn-expressing HMEC-1 cells, followed by extensive washing and incubation overnight. The amounts of IgA1 variants present in collected medium samples were quantified by ELISA. Shown as mean ± SD of triplicates from one representative biological replicate. ns > 0.05, ***P = 0.0001, ****P < 0.0001, by using unpaired t test. E) Plasma concentration (nM) at days 1, 3, and 7 of IgA1 and IgA1-DIII-QMP fusions. F) Log-linear changes in the plasma level (%) of IgA1 and DIII-QMP fusions in human FcRn transgenic mice. The IgA1 variants were administrated as a single i.v. injection to five mice per group. Shown as mean ±SD. ns > 0.05, *P = 0.0339, 0.0153, **P = 0.0017, ****P < 0.0001, by two-tailed analysis using unpaired t test.

In an attempt to solve this but to keep the fusion partner small in size, we took advantage of an engineered DIII with three amino acid substitutions in DIII (E505Q/T527M/K573P; QMP) (42). A full-length QMP-containing version has been shown to bind human FcRn with nearly 200-fold improved affinity at pH 5.5 and no binding at pH 7.4, which translated into more efficient cellular rescue and up to several-fold extended half-life in human FcRn transgenic mice when fused to coagulation factors (42, 47). Therefore, we here fused DIII-QMP to the C-terminal end of either the LCs or HCs of IgA1 (Figs. 4A and S1). This strategy resulted in similar HER2-binding profiles as unfused IgA1 (Fig. 4B and Table S1) and strong pH-dependent human FcRn binding (Fig. 4C). Accordingly, the QMP-containing DIII-fused IgA1 variants were shown to be efficiently rescued from intracellular degradation in HERA, whereas unfused IgA1 was not. Also, the LC fusion was rescued more efficiently than the HC fusion (Fig. 4D).

Next, we determined the plasma half-life of the DIII-QMP-fused AlbuAbs in human FcRn transgenic mice, which revealed that this strategy resulted in about 3-fold higher amounts in plasma at day 1 compared with unfused IgA1, increasing to a 7- to 9-fold difference on day 3 and up to 100-fold at day 7 (Fig. 4E). In line with the HERA results, the LC-fused variant showed a 1.2-fold longer plasma half-life than that of the HC-fused variant. This translated into an up to 4-fold extended plasma half-life (3.3 and 3.9 days) compared with unfused IgA1 (Fig. 4F), which was in line with values from the noncompartmental PK model, resulting in a 3.1- and 4.1-day plasma half-life for the HC- and LC-fused variants, respectively (Table S2). Accordingly, the AUC values for the DIII-QMP-fused variants were 6- to 7-fold higher than for naked IgA1 and the DIII-fused versions, with slower clearance values that were still 6- to 10-fold faster than for the full-length HSA-fused IgA variants (Table S2).

### DIII-QMP-fused IgA1 engages FcαRI favorably and induces potent ADCC

**Fig. 5.**
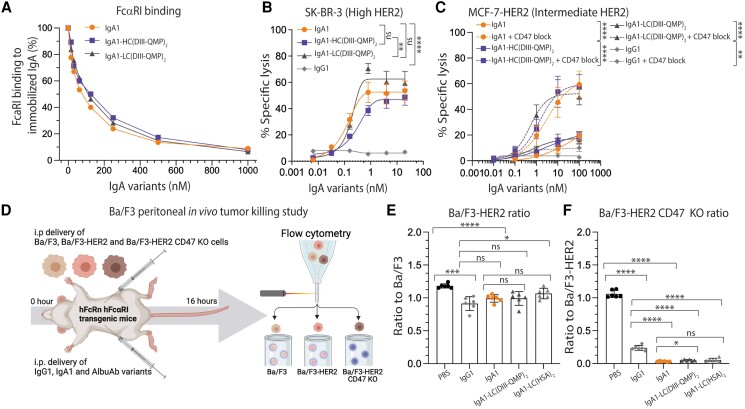
Albumin-equipped IgA1 variants engage FcαRI and mediate potent tumor killing in vitro and in vivo. A) Competitive binding of constant amount of His-tagged human FcαRI (100 nM) alone or with titrated amounts of IgA1 and IgA1-DIII-QMP (15.6–1,000.0 nM) over immobilized IgA1. B and C) ADCC assay showing the cytotoxic potential of IgA1 compared with that of the IgA1-DIII-QMP fusions in the presence of PMNs (effector:target cell [*E*:*T*] ratio = 40:1). The cytotoxic potential was studied both in the presence (dotted line) and absence (solid line) of CD47 blockade for all three variants. Specific lysis (%) of (B) SK-BR-3 and (C) MCF-7-HER2 was determined. Shown as means ± SEM of triplicates from three independent experiments performed using PMNs from three different donors. ns > 0.05, **P = 0.0017, 0.0011, ****P < 0.0001, by two-way ANOVA. D) Illustration of the in vivo tumor killing setup performed on mice transgenic for both the human forms of FcαRI and FcRn. The figure was made with BioRender. E) Ratio of Ba/F3-HER2 cells to Ba/F3 cells and the (F) ratio of Ba/F3-HER2 CD47 KO cells to Ba/F3-HER2 cells in mice treated with anti-HER2 IgG1, IgA1 compared with that of IgA1 variants fused to either HSA or DIII-QMP via the LCs. The test articles were administrated as a single i.p. injections to 6 mice per group. Shown as mean ±SD. ns > 0.05, * *P* = 0.0104, 0.0177, **P = 0.0017, ***P = 0.0001, ****P < 0.0001, by two-tailed analysis using unpaired t test.

To investigate the effect of fusing engineered DIII-QMP to IgA1 on the ability to bind FcαRI, the competition assay was performed as before (Fig. 2A), where the fusions were shown to compete well for binding to the receptor (Fig. 5A). We next measured their ability to lyse target cells with high (SK-BR-3, Fig. 5B) and intermediate (MCF-7-HER2, Fig. 5C) HER2 expression levels (Fig. S3). At high surface expression of HER2, both DIII-QMP fusions demonstrated potent ADCC capacity with no significant difference to that of IgA1. However, a difference was measured between the two DIII-QMP-fused formats, where the LC-fused mediated higher levels of lysis (Fig. 5B). In the presence of intermediate HER2 density (MCF-7-HER2), lower levels of lysis were measured, with 20% lysis capacity measured at the highest tested concentrations for all variants (Fig. 5C). ADCC capacity was also studied when targeting cancer cells with low HER2 levels (MCF-7, Fig. S3), where no activity was demonstrated (Fig. S4A). Furthermore, the effect of CD47 blockade was studied for cells expressing intermediate HER2 levels (Fig. 5C), which revealed a dramatic ADCC enhancement for both unfused and DIII-QMP-fused IgA1 (Fig. 5C). CD47 blockade was also studied for low HER2-expressing cells, where no enhancement was observed (Fig. S4A).

### In vivo tumor killing in a double transgenic mouse model

To investigate whether the in vitro antitumor activity translated into eradication of HER2-positive tumor cells in vivo, we took advantage of a new engineered mouse strain expressing the human forms of FcRn and FcαRI. The mice were used for a short syngeneic peritoneal model by intraperitoneal injection of HER2-transfected Ba/F3 cell lines, as described (12), either expressing or not expressing CD47 (Ba/F3, Ba/F3-HER2, or Ba/F3-HER2 CD47 knockout [KO]). IgA1 fused via the LCs to full-length HSA or DIII-QMP, showing the most efficient target cell killing in vitro and the longest plasma half-lives in vivo (Figs. 1J, 2D and E, 4F, and 5B and C) were intraperitoneally injected and benchmarked against unfused IgA1 and IgG1. After 16 h, Ba/F3, Ba/F3-HER2, and Ba/F3-HER2 CD47 KO cells were quantified by flow cytometry, and the ratios of Ba/F3 vs. Ba/F3-HER2 or Ba/F3 CD47 KO determined (Fig. 5D). The resulting Ba/F3 to Ba/F3-HER2 ratio revealed that all antibodies reduced the number of HER2-expressing tumor cells by 10–23% relative to the phosphate-buffered saline (PBS) group. No difference was measured between IgA1 and the two AlbuAbs formats (Fig. 5E). In contrast, a dramatic reduction in HER2-expressing cells KO for CD47 was measured, where treatment with IgA1 and the AlbuAbs eliminated almost all cells, and significantly better than treatment with anti-HER2 IgG1, which gave a 70–80% reduction (Fig. 5F). Quantification of the effector cells in the peritoneum revealed that all mice receiving antibody treatment had an increased number of neutrophils (Fig. S5).

### Albumin engineering of IgA1 has limited effects on *N*-glycosylation

Compared with IgG1, IgA is extensively glycosylated with both *O*- and *N*-linked glycosylation. In addition to the long hinge with 12 potential *O*-glycosylation sites, IgA1 has two conserved *N*-glycosylation sites, one located in the C_H_2 domain (N144) and one in the tailpiece (N340). Interestingly, IgA has been reported to be cleared from circulation by binding to the asialoglycoprotein receptor, expressed by hepatocytes of the liver and other tissues, which seems to be related to specific glycan structures (48, 49), where terminal galactose and low levels of sialic acid facilitate interaction with the receptor (12, 19, 49, 50).

To assess whether the observed effect on IgA1 half-life extension, when fused to full-length HSA or DIII-QMP could be explained by altered glycosylation, glycoproteomic analysis by liquid chromatography–mass spectrometry was performed. The results revealed that all the IgA1-based designs mostly contained complex diantennary-type *N*-glycosylation with high galactosylation (∼70% of antennae), partial sialylation (∼50% of galactoses), and very low fucosylation (∼10%; Fig. S6). The main differences between the fusions were found in the glycan complexity, where both IgA1-LC(HSA)_2_ and IgA1-LC(DIII-QMP)_2_ carried lower complexity *N*-glycan species than their HC counterparts and the unfused IgA1. For instance, whereas unfused IgA1 contained 76% complex species, 20% hybrid species, and 3% high-mannose species, IgA1-LC(DIII-QMP)_2_ showed respective percentages of 62, 33, and 5%. Furthermore, IgA1-LC(HSA)_2_ was measured with the lowest complexity across the fusions, with only 45% complex, 34% hybrid, and 21% high-mannose species.

While IgA1-LC(DIII-QMP)_2_ and especially IgA1-LC(HSA)_2_ showed relatively low levels of uncapped galactoses, which should be beneficial for plasma half-life, the hybrid and high-mannose species would be expected to have negative effects on persistence (51). However, as HSA and DIII-QMP-fused variants consistently showed slower clearance than the unfused IgA1, it was apparent that the *N*-glycosylation properties resulting from the engineering processes were not a significant factor in determining half-life differences as observed in vivo.

### QMP-DIII-fused IgA2 mediates the killing of HER2- and CD20-expressing cancer cells

**Fig. 6.**
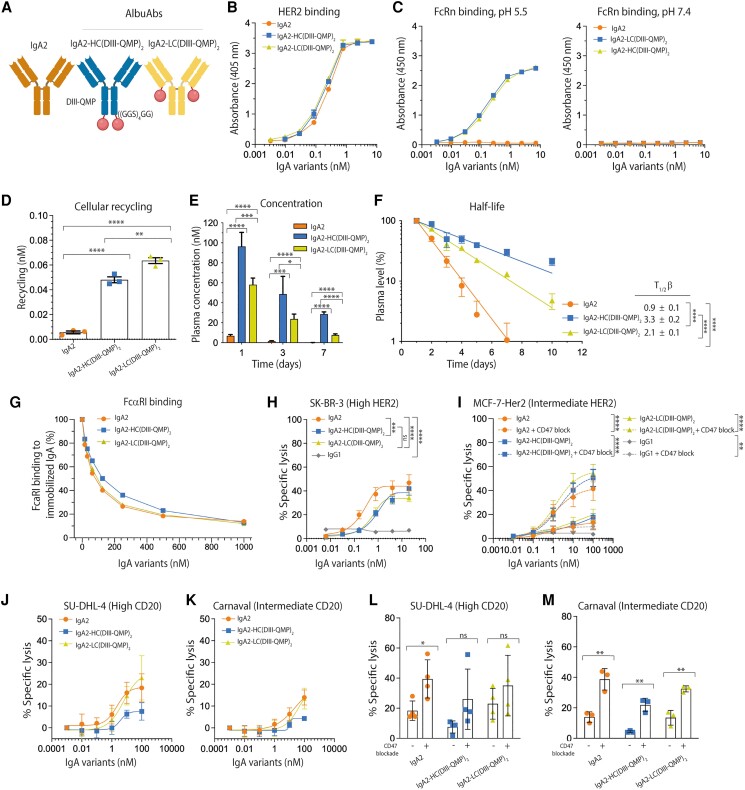
IgA2-fused DIII-QMP shows extended plasma half-life and ADCC activity. A) Schematic illustration of IgA2 and DIII-QMP genetically fused either the HCs or LCs of human IgA2 via a flexible glycine–serine linker ((GGS)_4_GG). The tailpiece of the IgA2 variants was deleted. The figure was made with BioRender. B) ELISA results showing binding of titrated amounts of IgA2 and IgA2-DIII-QMP fusions to recombinant HER2 coated in wells at pH 7.4. Shown as mean ± SD of duplicates. C) ELISA results showing binding of GST-tagged human FcRn at pH 5.5 and 7.4 to titrated amounts of IgA2 and DIII-QMP-fused variants captured on HER2 coated in wells. Shown as mean ± SD of duplicates. D) 400 nM of each of IgA2 and IgA2-DIII-QMP variants were added for 4 h to human FcRn-expressing HMEC-1 cells, followed by extensive washing and incubation overnight. The amounts of IgA2 variants present in collected medium samples were quantified by ELISA. Shown as mean ± SD of triplicates from one representative biological replicate. ns > 0.05, **P = 0.0097, ****P < 0.0001, by using unpaired t test. E) Plasma concentrations (nM) at days 1, 3, and 7 of IgA2 and IgA2-DIII-QMP fusions. (F) Log-linear changes in the plasma level (%) of IgA2 and DIII-QMP fusions in human FcRn transgenic mice. The IgA2 variants were administrated as a single i.v. injection to five mice per group. The data are represented as mean ±SD. ns > 0.05, *P = 0.0172, ***P = 0.0011, 0.0004, 0.0006, ****P < 0.0001, by two-tailed analysis using unpaired t test. G) Competitive binding of constant amount of His-tagged human FcαRI (100 nM) alone or with titrated amounts of IgA2 and IgA2-DIII-QMP (15.6–1,000.0 nM) over immobilized IgA2. H and I) ADCC assay showing the cytotoxic potential of IgA2 compared with that of the IgA2-DIII-QMP fusions in the presence of PMNs (effector cell:target cell [*E*:*T*] = 40:1), in the presence (dotted lines) and absence (solid lines) of CD47 blocking. Specific lysis (%) of (H) SK-BR-3 and (I) MCF-7-HER2 cells was determined. Shown as mean ± SEM of triplicates from three independent experiments with PMNs from different donors. ns > 0.05, **P = 0.0024, ***P = 0.0001, ****P < 0.0001, by two-way ANOVA. J and K) ADCC assay showing the cytotoxic potential of IgA2 compared with that of the IgA2-DIII-QMP fusions in the presence of PMNs (effector cell:target cell [*E*:*T*] = 40:1). Specific lysis (%) of (J) SUDHL-4 and (K) Carnaval cells was determined. Shown as mean ± SEM of triplicates from three independent experiments with PMNs from different donors. L and M) Specific lysis (%) of (L) SUDHL-4 and (M) Carnaval cells was also determined, in the presence and absence of CD47 blockade. Shown as mean ± SEM of triplicates from four (SUDHL-4) or three (Carnaval) individual experiments with PMNs from different. L) ns > 0.05, *P = 0.0251, (M) **P = 0.055, 0.0024, 0.0032, by using unpaired t test.

Human IgA exists as two subclasses where IgA1 predominates in blood and IgA2 in secretions (52–55). Despite sharing 90% similarity in amino acid composition and equal ability to engage FcαRI, IgA2 has a truncated hinge region lacking 12 putative *O*-glycans and up to 3 additional *N*-glycan sites as found in IgA1 (56–59). Interestingly, *O*-glycosylated IgA1 has been linked to the pathogenesis of Berger's disease (60), which may make it less attractive as a scaffold in the design of therapeutics. Due to this, we extended the QMP-DIII concept to demonstrate that it could be applied to IgA2 with HER2 specificity (Figs. 6A and S7). The results showed that although having a shorter hinge, fusion to engineered DIII-QMP did not hamper HER2 or FcRn binding (Fig. 6B and C and Table S1). This further translated into improved FcRn-mediated recycling and extended plasma half-life of both designs compared to the parental IgA2 (Fig. 6D–F and Table S2). However, a distinct difference in half-life was revealed between the HC-fused (3.3 days) and LC-fused (2.1 days) DIII-QMP IgA2 variants (Fig. 6F and Table S2), which was in contrast to the opposite measured in HERA (Fig. 6D). The IgA2 designs were further shown to bind FcaRI and to engage PMNs for directed killing of HER2-expressing cancer cell lines (Fig. 6G–I), which was enhanced by CD47 blockade (Fig. 6I).

Finally, to address whether our strategy for half-life extension could be broadly applicable to IgA antibodies, we included the variable region of rituximab in an IgA2-DIII-QMP specific for CD20 (Fig. S7D–F). In this case, ADCC activity was studied in the presence of PMNs and human lymphoma cell lines with high (SUDHL-4) or intermediate (Carnaval) surface expression of CD20 (Fig. S8). The results showed that the LC-fused DIII-QMP IgA2 performed equally well to that of the parental unfused IgA2, while the HC-fused IgA2 variant mediated slightly lower levels of lysis against both cell lines (Fig. 6J and K). When ADCC was performed in the presence of CD47 blockade, cell lysis was enhanced against both high and intermediate CD20-expressing cells (Fig. 6L and M). Again, no activity was demonstrated at low antigen density when using HER2-expressing cells (Fig. S4B).

In summary, fusion of DIII-QMP could be extended to IgA2 directed toward two distinct tumor antigens, where target cell killing could be potentiated by blocking of the myeloid SIRPα-CD47 axis.

## Discussion

We report on how monomeric human IgA can be engineered for favorable PK properties combined with the ability to engage FcαRI expressed by PMNs for efficient killing of targeted cancer cells. Regarding half-life extension, this was achieved by taking advantage of albumin and its interaction with FcRn, which acts as a half-life controller of albumin (30, 31, 34). To do so, we introduced two FcRn-binding sites on IgA by fusing a full-length HSA or a derived DIII fragment to either LCs or HCs. This rationale was based on the fact that FcRn binds homodimeric IgG with a 2:1 stoichiometry with identical affinities at the two independent C_H_2–C_H_3 elbow-binding sites (29, 61–63) and that both binding sites are known to be required for efficient IgG rescue from intracellular degradation (63). In stark contrast, FcRn binds albumin in a 1:1 fashion (30, 34). We speculated whether mimicking the avidity of IgG could be an advantage to achieve FcRn engagement of HSA-equipped IgA, even though the spatial distance would not be identical.

To test the concept, we used a trastuzumab-based IgA and genetically fused full-length HSA (66.5 kDa) or only its C-terminal DIII (23 kDa) to the LCs or HCs via a flexible glycine–serine linker. Both strategies resulted in fusion formats with expected molecular weights that were well produced in a human cell expression system. Importantly, when adding a fusion partner to IgA, its ability to engage FcαRI via each side of the C_H_2–C_H_3 elbow region should not be abolished (64). Indeed, our results show that this is the case for the designed IgA1 fusions and that FcαRI-expressing PMNs can be engaged and induce ADCC against HER2-expressing cancer cells. However, fusion of both full-length HSA and DIII-QMP reduced binding to FcαRI in vitro, where full-length HSA showed the most pronounced effect. This steric effect likely explains the reduced ADCC activity measured for the larger full-length HSA IgA fusion. In addition, the LC fusions showed more similar PMN-induced ADCC activity to that of unfused IgA1 followed by the HC fusions, which was the case for both the full-length HSA and DIII-QMP-fused IgA variants.

The concept was also extended to human IgA2, where fusion of DIII-QMP to the LCs and HCs was shown to not negatively affect engagement of FcαRI. This was the case despite the fact that IgA2 has a shorter hinge region than that of IgA1. However, IgA2 with fusion of DIII-QMP to its LCs showed almost 2-fold shorter plasma half-life than the corresponding IgA1 variant in human FcRn-expressing mice, which is in contrast to the half-life values for the HC fusions of the two subclasses that were similar. The reason for this is not known, but we speculate that the less flexible hinge of IgA2 in combination with the fusion of DIII-QMP to the LCs may cause steric hindrance or an orientation of DIII-QMP that is less favorable for FcRn engagement in vivo, despite the fact that such an effect was not measured for direct binding to human FcRn or in HERA. Alternatively, factors that are FcRn independent contribute to this discrepancy.

The versatility of the AlbuAbs technology in an IgA format was demonstrated for HER2 targeting but also CD20. In addition, we showed that ADCC could be boosted by combining the albumin-equipped IgA1 and IgA2 designs by blocking the myeloid SIRPα-CD47 checkpoint.

While reduced ADCC activity was measured in vitro for IgA1 fused to full-length HSA, both the LC- and HC-fused formats were efficiently rescued from cellular degradation in HERA, and in vivo in human FcRn-expressing mice. This resulted in a >7-fold extended plasma half-life and a dramatic increase in availability as the amounts present in blood were up to 10-fold higher than that measured for naked IgA1. In contrast, when IgA1 was fused to DIII-WT, at either the LCs or HCs, the formats were not rescued by FcRn, which gave a 1-day plasma half-life, similar to that of naked IgA1.

We based our strategy on DIII as it harbors the principal binding site for human FcRn (30, 34, 40). However, we have also shown that the N-terminal DI of albumin contributes to optimal receptor binding (40, 46). Hence, recombinant DIII-WT binds with 10-fold reduced affinity in the absence of DI compared with full-length HSA (30, 40), and we here show that this loss of binding cannot be compensated for by fusing two DIII molecules to one IgA1 molecule. To overcome this, we introduced the QMP substitutions (42) in DIII, which allowed both IgA1 and IgA2 fusions to bind FcRn favorably, resulting in up to 4-fold extended plasma half-life in human FcRn-expressing mice. Notably, the DI contribution to FcRn binding is species specific as recombinant mouse DIII binds very similarly to that of full-length mouse albumin (40).

Structurally, 25% of the contact surface with human FcRn is lost when only DIII is used (albumin:FcRn = 1,815 Å^2^, DIII:FcRn = 1,373 Å^2^), which also exposes the N-terminal end, one α-helix (residues 441–467), and a loop (479–483) of DIII (Fig. S9). This does not only reduce the surface interacting with the receptor but also exposes residues that usually are buried within full-length HSA. However, most of these residues are nonhydrophobic, and we therefore expect that this will not cause aggregation, which is in line with the monomeric nature of the purified fractions. Whether the engineered DIII-IgA designs could trigger an immune response against surfaces that are normally shielded by the two other domains of HSA is unknown, but we assume that this is unlikely due to its human origin.

Previously, efforts have been made to combine structural elements of IgA and IgG (65–68) with the aim of engaging both FcαRI and FcRn. This has been explored by engineering the C_H_2 domain of IgG by replacing selected amino acid residues with the corresponding residues of IgA1 combined with fusion to the IgA1–C_H_3 domain (65). Such a hybrid format allowed for FcαRI engagement, but disrupted binding to human FcRn, which is not a surprise from a structural point of view, as both the C_H_2 and C_H_3 domains are required for IgA to bind FcαRI (69) and IgG to bind FcRn (29, 70). This pinpoints that engineering the FcαRI- and FcRn-binding sites into the same C_H_2–C_H_3 elbow region may not be straightforward, which further supports the strategy explored here where the FcRn-binding sites were introduced distally from the IgA-Fc elbow region. Designing FcαRI-binding molecules based on IgG will also allow for tailoring of additional effector functions, such as recruitment of the complement system or engagement of FcγRs (65–68).

Considering that injected antibody formats should reach the tumor site, plasma concentration and exposure time are important parameters. While IgA has a molecular size above the renal clearance threshold, it suffers from a limited plasma half-life compared with albumin and IgG antibodies that are rescued by FcRn. In this work, we show that fusion of IgA to full-length HSA or DIII-QMP resulted in 10- to 20-fold higher plasma concentrations than unfused IgA, which was the highest for the full-length HSA fusions that also showed 2-fold longer plasma half-life than the DIII-QMP fusions. Regarding the effective killing of solid tumors, the modalities should also penetrate the tumor tissue, which may be affected by molecular size and biophysical properties. In general, larger formats are expected to show more limited tumor penetration than smaller modalities due to poor diffusivity and permeability (71–73). Interestingly, reports support that albumin accumulates at cancer sites with vascular leakiness where it is metabolized as an energy source (74–77). However, the impact of FcRn expression in this context is far from fully understood. While loss of FcRn expression has been speculated to result in albumin accumulation (78), associated with poor prognosis (79, 80), another study reports that FcRn is up-regulated in cancer tissues and suggests that it drives albumin uptake and cancer growth (81). Thus, HSA-based anticancer designs may have beneficial properties regarding tumor targeting and reach of the tumor microenvironment, which should be addressed in future studies.

Interestingly, IgA has been reported to be cleared by binding to the asialoglycoprotein receptor expressed by hepatocytes due to specific glycan structures attached to IgA (48, 49). However, this route of clearance is overcome when introducing FcRn targeting, as in this study, and shown by indirect FcRn targeting of endogenous albumin, as reported (19, 44). Accordingly, mapping of the *N*-glycosylation patterns of the albumin-equipped IgA formats revealed small but distinct differences, but these could not be correlated to the variation in plasma half-lives measured between the formats. Notably, the complexity of glycosylation may be a challenge for scale-up manufacturing of IgA (58), which could be overcome by glycoengineering strategies, as reviewed (9).

Another factor that has limited preclinical studies of IgA is that mice do not express an orthologue of human FcαRI (82). As a result, its therapeutic potential has been difficult to investigate, and the focus has been on IgG as a scaffold. However, the combination of transgenic mice expressing the human receptor (83) with in vitro human cellular assays (11, 13, 14) has provided a growing body of evidence supporting that monomeric human IgA is effective in eradicating tumor cells. However, in vivo studies of human IgA in mice have been hampered by its short plasma half-life compared with IgG, which further requires higher and more frequent dosing of IgA. In addition, due to cross-species FcRn-binding differences, Fc-engineered human IgG- and HSA-based strategies must be conducted in mice transgenic for human FcRn and KO for the mouse counterpart (36–42). Thus, we performed all PK studies in such mice.

To further verify the concept of albumin-equipped anticancer IgA in vivo, we have engineered a mouse strain that is transgenic for both the human forms of FcRn and FcαRI. Despite that IgA1 fused to full-length HSA showed reduced ADCC activity in vitro, this was not the case in mice given HER2-expressing cancer cells, where both the LC-fused HSA and DIII-QMP IgA1 variants showed a similar cytotoxic effect to that of unfused IgA1. In addition, the AlbuAb formats performed better than the IgG1 counterpart, as IgA and the AlbuAbs more efficiently depleted the CD47 KO tumor cells. These encouraging results motivate further in vivo studies on the long-acting AlbuAb formats.

Beyond cancer, not only secretory IgA but also monomeric IgA may be attractive for treatment of infectious diseases, as demonstrated for SARS-CoV-2 (84, 85). However, to be used prophylactically, IgA will benefit from being engineered for prolonged plasma half-life. While we have previously demonstrated that the half-life of IgA can be extended by fusion of a bacterially derived albumin-binding domain to IgA, which hijacks endogenous albumin post injection (19, 44), such a strategy raises concerns regarding immunogenicity (44). An alternative could be to take advantage of a heterodimeric IgA-Fc platform to design bispecific IgA molecules where one arm targets the FcRn recycling pathway (86). Such a strategy can be combined with technologies allowing correct HC and LC pairing of the two specificities. A bispecific IgA could, for instance, be engineered to bind albumin with one arm without interfering with pH-dependent FcRn engagement. However, such indirect targeting of FcRn will be limited to the plasma half-life of the complex with endogenous albumin itself. Thus, we provide here a strategy where either full-length HSA or an engineered DIII can be fused directly to either of the two human IgA subclasses to gain favorable PK properties. This strategy should also be attractive for half-life extension of other antibody modalities, including other isotypes and non-Ig formats.

## Materials and methods

### Study design

The objective of this study was to design an albumin-based half-life extension technology to be combined with IgA with cancer specificity. The aim was to have resulting IgA molecules that would be able to interact and be recycled by FcRn through the genetically fused albumin domains that would not interfere with antigen and FcαRI binding, as well as have the ability to eliminate a target cancer cell. For interaction and cellular studies (ELISA, SPR, HERA), the sample size was determined based on our experience with studies on IgG and albumin, and performed three times. Studies involving effector cells from blood isolated from healthy donors were performed at University Medical Center (UMC) Utrecht and Christian-Albrecht University (CAU) in Kiel, approved by the ethical committees of the participating institutions in accordance with the Declaration of Helsinki (UMC Utrecht: Medical ethical approval protocol 07-125/O, CAU Kiel: D 557/16). Before blood donation, volunteers signed a written informed consent form giving permission to use their blood for research purposes. Mouse studies performed in collaboration with The Jackson Laboratory were approved by their internal Animal Care and Use Committee, and acted in accordance with European standards of research ethics and the Declaration of Helsinki. Five mice per group were used, which is the standard number of animals used to determine IgG half-life and which allow for statistical analysis. The in vivo studies were performed once, due to ethical considerations and animal welfare.

### Cell culture

The HEK293E cell line (ATCC, catalog number: CRL-1573) was grown in RPMI medium (Merck, catalog number: R2405) added 10% heat-inactivated fetal calf serum (FCS; Merck, catalog number: F7524), and 1% Penicillin–Streptomycin (Merck, catalog number: P4458). The high five cell line (Invitrogen, catalog number: B85502) was kept in Express FIVE SEF medium (ThermoFisher, catalog number: 10486025) added 1% antibiotic-antimycotic (ThermoFisher, catalog number: 15240096) and 18 mM L-glutamine (ThermoFisher, catalog number: 25030123). The human microvascular endothelial cell-1 (HMEC-1) cell line (87) was grown in Gibco MCDB 131 medium (ThermoFisher, catalog number: 10372019) added 10% heat-inactivated FCS (Merck, catalog number: F7524), 10 ng/mL mouse epidermal growth factor (ThermoFisher, catalog number: PMG8043), 1% Penicillin–Streptomycin (Merck, catalog number: P4458), 1 µg/mL hydrocortisone (Merck, catalog number: H0888), and 2 mM L-glutamine (ThermoFisher, catalog number: 25030123). To maintain stable FcRn expression, the medium was added 5 µg/mL blasticidin (ThermoFisher, catalog number: A1113903) and 100 µg/mL G418 (ThermoFisher, catalog number: 10131027) (33).

The SK-BR-3 (ATCC, catalog number: HTB-30) and MCF7 (ATCC, catalog number: HTB-22) cell lines were cultured in RPMI medium (ThermoFisher, catalog number: 61870036) supplemented with 10% heat-inactivated FCS (Sigma, catalog number: F7524) and 1% Penicillin–Streptomycin (Gibco, catalog number: 11528876). The MCF7-HER2 cell line was generated by retroviral transduction with human HER2 (pMX-puro-HER2) followed by positive selection using puromycin.

The Carnaval and SU-DHL-4 cell lines were obtained from DSMZ (German Collection of Microorganisms and Cell Cultures). The cells were grown in RPMI Medium 1640 (1×) + GlutaMAX (Gibco/Thermo Fisher Scientific, catalog number: 72400-021) added 5% Pen Strep (Gibco/Thermo Fisher Scientific, catalog number: 15140-122), while the cell lines were cultured in 10 and 20% FBS (Gibco/Thermo Fisher Scientific, catalog number: 10270-106) containing RPMI, respectively.

### Antibody production and purification

cDNA encoding the variable regions of the HC and LC derived from trastuzumab in frame of human IgA1 and IgA2 were obtained in the expression vectors pEE14.4-kappaLC, pEE14.4-IgA1, and pEE14.4-IgA2(m1), as reported (19). cDNA encoding an engineered HSA-DIII fragment (E505Q/T527M/K573P (42)) was subcloned in frame with the C-terminal end of the HC or LC linked by a segment encoding a glycine–serine linker ((GGS)_2_GG). The segments encoding the tailpiece were removed by introduction of a stop codon. The IgA constructs designed to bind CD20 were designed by using the variable regions of the HC and LC of rituximab.

The HC and LC vectors encoding HER2 IgA subclasses and DIII fusions were transiently co-transfected into adherent HEK293E cells using Lipofectamine2000 (ThermoFisher, catalog number: 10600714). Growth medium was harvested and replaced every day for 2 weeks prior to purification using a CaptureSelect IgA affinity matrix (ThermoFisher, catalog number: 1942880250) packed column (Atoll), as described by the manufacturer. The collected IgA variants were concentrated, and the buffer was changed to PBS (Merck, catalog number: D8537) using Amicon Ultra-15 mL 50 K columns (Millipore, catalog number: UFC905024) before SEC using a Superdex 200 increase 10/300GL column (GE Healthcare, catalog number: 28-9909-44) coupled to an ÄKTA Avant instrument (GE Healthcare). Monomeric fractions were collected and concentrated using Amicon Ultra-0.5 mL 100 K columns (Millipore, catalog number: UFC510024) and analyzed on a Superdex 200 increase 3.2/300 column (GE Healthcare, catalog number: 28990946) coupled to an ÄKTA HPLC instrument (GE Healthcare).

### Recombinant human FcRn production and purification

A vector encoding soluble recombinant GST-tagged human FcRn (pcDNA3-GST-hβ2-microglobulin) created from a pcDNA3.1 vector (Invitrogen, catalog number: V79020) was transiently transfected into HEK293E cells. Secreted receptor was harvested for up to 2 weeks before collected supernatant was purified using a GSTrap HP column (GE Healthcare, catalog number: 17-5282-02), all as described previously (88). Soluble recombinant His-tagged human FcRn was produced with a Baculovirus expression vector system (89) (the viral stock was a gift from Dr Sally Ward, University of Southampton, United Kingdom). Harvested supernatant containing secreted receptors was further purified with a HisTrap HP column supplied with Ni^2+^ ions (GE Healthcare, catalog number: 17-5248-02) (90).

### Antigen binding

Ninety-six-well ELISA plates (Costar, catalog number: 10544522) were coated with 1.0 μg/mL recombinant human HER2 (Sino Biological, catalog number: 10004-HCCH) diluted in PBS (Merck, catalog number: D8537), and incubated ON at 4 °C. Next, the wells were blocked for 1 h at room temperature (RT), using PBS with 4% skimmed milk (S) (ITW reagents, catalog number: A0830). The plates were washed four times with PBS containing 0.05% Tween 20 (T) (Merck, catalog number: P1379) before 100 μL of titrated amounts of IgA and IgA-DIII variants in PBS/S/T were added, and incubated for 1 h on a shaker at RT. The plates were then washed, and 100 μL of an alkaline phosphatase (ALP) conjugated antihuman IgA (α-chain specific) antibody produced in goat (Merck, catalog number: SAB3701226) was added (1:2,000) and incubated for 1 h at RT. After washing, bound proteins were detected by adding 100 μL of ALP substrate (1 mg/mL phosphate in diethanolamine buffer; Merck, catalog number: S0942). Absorbance was measured at 405 nm with a Sunrise spectrophotometer (TECAN). In addition to the setup as described being performed at pH 7.4, binding to HER2 was also measured at pH 7.0, 6.5, and 6.0 by adjusting the pH of the PBS/S/T.

### Human FcRn binding

Ninety-six-well ELISA plates (Costar, catalog number: 10544522) were coated, blocked, and added titrated amounts of IgA and IgA-DIII variants as above, before the wells were washed with PBS/T pH 5.5 or pH 7.4, and added 2 μg/mL of human FcRn-GST in PBS/S/T pH 5.5 or 7.4. After incubation for 1 h at RT, the wells were washed with pH 5.5 or 7.4, and added 100 μL horseradish peroxidase conjugated goat anti-GST antibody (Rockland, catalog number: 600-102-200), diluted (1:8,000) in PBS/S/T with pH 5.5 or 7.4 before incubation for additional 1 h at RT. Plates were washed, and 100 μL of 3,3′,5,5′-tetramethybenzidine substrate solution (Merck Millipore, catalog number: CL07) added. Absorbance was measured at 620 nm with a Sunrise spectrophotometer (TECAN), and the reaction terminated by adding 50 μL 1 M HCl, and then measured again at 450 nm.

### Human FcαRI binding

Ninety-six-well ELISA plates (Costar, catalog number: 10544522) were coated, blocked and incubated with IgA and IgA-DIII variants as above followed by 100 μL of His-tagged human FcαRI (Sino Biological, catalog number: 10414-H08H), at a concentration of 2 µg/mL and incubated for 1 h at RT. The plates were washed before 100 μL of an ALP-conjugated anti-His-tag antibody (Abcam, catalog number: ab49746) was added (1:5,000) and incubated for 1 h at RT. The plates were washed, and bound proteins detected by 100 μL of ALP substrate, and absorbance was measured at 405 nm with a Sunrise spectrophotometer (TECAN).

### Surface plasmon resonance

A Biacore T200 (GE Healthcare) instrument was used following the manufacturer's protocol. Competitive binding experiments were performed by injecting a constant amount of His-tagged human FcαRI (100 nM) alone or mixed with IgA, IgA-DIII, or IgA-HSA variants (15.6–1,000.0 nM) over immobilized IgA1 or IgA2 (150 RU). HBS-P + buffer (20 mM Tris-HCl, 150 mM NaCl, 0.005% Surfactant P20) at pH 7.4 was used as the running buffer. All binding curves were zero adjusted, and the reference flow cell value was subtracted. Binding response of FcαRI to IgA in the absence of a competitor was set to 100%.

### Human PMN ADCC assays

Analysis of ADCC on HER2-expressing cells was measured using a chromium-release assay, as described (13, 19). Briefly, SK-BR-3 (ATCC, HTB-30) or MCF7-HER2 cells were labeled for 2 h with 100 µCi (3.7 MBq) ^51^Cr and were added to titrated amounts of IgA and HSA/DIII/DIII-QMP-fused IgA variants in round-bottom microtiter plates (Corning Inc, catalog number: 3799). CD47 was blocked by using a SIRPα IgG1 PGLALA fusion protein that was produced and purified in-house, as described previously (91). Tumor cells were pretreated with 10 μg/mL SIRPα IgG1 PGLALA fusion protein for at least 30 min at RT. PMNs were isolated from the blood of healthy donors (MiniDonorDienst UMC Utrecht) using a ficoll-histopaque density gradient and added with an *E*:*T* = 40:1 in a volume of 200 μL/well before incubated for 4 h at 37 °C. Next, the supernatant was transferred to a LumaPlate (Perkin Elmer, catalog number: 6006633) and counted in a liquid scintillation counter (in cpm) (Perkin Elmer). Lysis was calculated using the formula: %lysis=([Experimentalcpm−Basalcpm]/[Maximalcpm−Basalcpm])×  100. Background levels were determined with culture medium only, and 3% Triton-X-100 (Merck, catalog number: T8787) was used to determine maximum release.

Analysis of ADCC on CD20-expressing cells was performed by [^51^Cr] (Hartmann Analytic GmbH, catalog number: Cr-RA-8-5) release assay, as previously described (92). Peripheral blood was collected from healthy informed volunteers in S-Monovette (8.2 mL 9NC; Sarstedt, catalog number: 01.1606.001). Human PMN effector cells were isolated from human peripheral blood using Polymorphprep (Progen, catalog number: 1114683) as previously described (1). GM-CSF (50 U/mL, Cellgenix GmbH, catalog number: 1412-010) stimulated PMN, antibodies (0.01–100 nM) and medium were added to round-bottom microtest plates (Sarstedt, catalog number: 82.1582.001). CD47 was blocked by using anti-CD47 IgG2 FcKO blocking antibody (93). Assays were started by addition of effector and target cells at *E*:*T* ratio 40:1 (200,000:5,000). After 3 h at 37 °C, cell pellets were centrifuged, and [^51^Cr] release from triplicate samples was measured by transferring the supernatant (25 µL) into the scintillation reagent Optiphase HiSafe 3 (150 µL; Perkin Elmer, catalog number: 1200-437) in flexible-96 Clear Microplate (PerkinElmer, catalog number: 1450-401). Radioactive signal was measured with the MicroBeta TriLux 1450 Liquid Scintillation Counter (PerkinElmer). The specific lysis was calculated by using the formula as above. Maximal release of [^51^Cr] by tumor cells was achieved by adding Triton-X-100 (2% v/v), and the basal release was measured in the absence of antibodies and presence of stimulated effector cells.

### Quantification of target cancer cell antigen levels

HER2 expression level per cell was determined using a QIFIKIT (Agilent/Dako, catalog number: K007811-8), according to the manufacturer's instructions. The SK-BR-3, MCF7-HER2 and MCF-7 cell lines were stained with a saturating concentration of 10 mg/mL unconjugated mouse IgG1 monoclonal antibody directed against HER2 (Clone: 24D2, BioLegend, catalog number: 324402) followed by detection using F(ab′)2 fragment of fluorescein isothiocyanate (FITC)-conjugated goat antimouse Igs (Agilent/Dako, catalog number: K007811-8). Antihuman CD20 mouse IgG2 antibody (BioLegend) was used together with FITC-conjugated goat antimouse Fc-specific F(ab)2 fragments to detect CD20 expression on the Carnaval and SU-DHL-4 cell lines. Measurements were performed on a BD FACS Canto II.

### Human endothelial cell–based recycling assay

A HERA, using an HMEC-1 cell line stably expressing HA-hFcRn-EGFP, was performed as reported (33). One day before the experiment, 7.5 × 10^4^ cells/well were seeded into 24-well plates (Costar, catalog number: 10732552) and cultured in complete growth medium. Next day, the cells were washed and starved in Hank's balanced salt solution (HBSS; ThermoFisher, catalog number: 14025100). After 1 h of starvation, 250 µL of 400 nM of IgA variants diluted in HBSS (pH 7.4) were added to the cells and incubated for an additional 4 h. The medium was then removed, cells were washed with ice-cold HBSS (pH 7.4) and growth medium (without FCS) supplemented with MEM nonessential amino acids (ThermoFisher, catalog number: 11140050) were added. The cells were incubated ON, and samples were collected the next day. IgA and IgA-DIII present in the medium at the end of the experiment were quantified using ELISA.

### In vivo half-life studies

Mouse studies, conducted at The Jackson Laboratory (JAX Services, Bar Harbor, ME), were approved by their Animal Care and Use Committee and performed in accordance with guidelines and regulations. The studies were performed using homozygote Tg32 mice expressing human FcRn instead of the mouse counterpart and not mouse albumin (B6.Cg-Tg(FCHR)*32Dcr Alb*^em12Mvw^  *Fcgrt*^tm1Dcr^/MvwJ). Each test article was studied in groups of five mice. All mice were male, aged 6–10 weeks, with a weight between 18 and 31 g. The mice received 1 mg/kg of the IgA variants diluted in PBS, which were given by intravenous injections. Blood samples (25 µL) were collected from the retro-orbital sinus at days 1, 2, 3, 4, 5, 7, 10, 12, 16, 19, and 23 after injection. Plasma was immediately isolated from the blood samples by mixing with 1 µL 1% K3-EDTA and centrifuged at 17,000 × *g* for 5 min at 4 °C and stored at −20 °C until analysis. For quantification in ELISA, the samples were diluted 1:50–1:400 in PBS/S/T, and 100 μL was added per ELISA well, using individual standards. PK parameters were determined based on the measured plasma antibody concentrations using gPKPDsim for MatLab (43).

### Half-life calculation

The half-life of the IgA and DIII- and HSA-fused variants in plasma is presented as a percentage remaining, where 100% is the concentration at day 1. Prism 8 was used to perform linear regression analysis that drew a straight line through the data. β-Phase half-life was calculated by: $t_{1/2}=\;(\log\;\;0.5)/(\log\;Ae/A0)\times \;t$ (*t*_1/2_ = half-life, *Ae* = amount left, *A*0 = amount on day 1, *t* = elapsed time), as previously described (94). The *t*_1/2_ of the elimination phase for the test articles was determined using data points between days 3 and 23 post injection.

### MS for *N*-glycosylation analysis

The mass spectrometry analysis to map the *N*-glycan structures attached to the recombinant IgA variants was performed essentially as previously described (22).

### Generation of hFcRn hFcαRI transgenic mice

Mice were housed and bred at Janvier Labs (France). Human FcαRI transgenic mice (83) were crossed with CB-17/Icr-Prkdc^scid/sscid^ mice resulting in FcαRI transgenic mice on a CB-17 SCID background. These mice were crossed with stock 031644 (B6.Cg-Tg(FCGRR)*32Dcr Alb*^em12Mvw^  *Fcgrt*^tm1Dcr^Prkdc^scid^/J, The Jackson Laboratory), resulting in mice transgenic for FcαRI, and also for human FcRn instead of the mouse counterpart (homozygote Tg32 mice) and were hemizygous KO for mouse albumin. Mice were transferred to our animal facility 1 week before the study, and food and water were given ad libitum. The mice were housed in groups under a 12:12 light–dark cycle. For experiments, 10- to 38-week-old males were used and randomized based on age and genotype, and researchers were single blinded. The experiments were approved by the institute's animal ethics committee and by the Dutch Central Authority for Scientific Procedures on Animals (CCD, AVD11500202115442).

### Ba/F3 peritoneal model

The Ba/F3 peritoneal model was performed as described (12). Briefly, Ba/F3, Ba/F3-HER2, or Ba/F3-HER2 CD47 KO cells were labeled with 1 µM CFSE (eBioscience, catalog number: 65-0850-84) 5 or 0.5 µM cell trace violet (Invitrogen, ThermoFisher, catalog number: 10220455) for 15 min at RT and mixed at 1:1:1 ratio. In total, 1.5 × 10^7^ cells were injected intraperitoneally per mouse in 300 µL PBS. One hundred micrograms of each antibody (*n* = 6 mice per compound) were injected intraperitoneally after the injection of tumor cells. Sixteen hours later, the mice were euthanized followed by washing of the peritoneum with PBS containing 5 mM EDTA. The absolute number of Ba/F3, Ba/F3-HER2, or Ba/F3-HER2 CD47 KO was determined by flow cytometry using TruCount tubes (BD Biosciences, catalog number: 663028), and the ratio of Ba/F3 and Ba/F3-HER2 or Ba/F3 CD47 KO calculated. Effector cells in the peritoneum were determined using specific antibodies, and their relative amounts were related to the constant number of beads (Invitrogen, catalog number: 11550696).

### Antibodies and flow cytometry

Effector cells in the peritoneum were determined after incubation with 5% normal mouse serum (Equitech-Bio, catalog number: MS05) for 45 min at 4–7 °C. Subsequently, the following fluorescently labeled antibodies were used for 45–60 min at 4–7 ^o^C to determine the different effector cells types: FcαRI (A59) (BioLegend, catalog number: 354109), Ly-6G (1A8) (BioLegend, catalog number: 127618), FcγRIV (CD16.2, 9E9) (BioLegend, catalog number: 149531), Siglec-F (S17007L) (BioLegend, catalog number: 155509), F4/80 (BM8) (BioLegend, catalog number: 123118), and CD11b (m1/70) (eBioscience, catalog number: 17-0112-80). Measurements were performed on a FACSCantoII (BD Biosciences), and the results analyzed using FACS Diva software (BD Biosciences).

### Interaction surface analysis

The interaction surface analysis was done using the HSA-FcRn complex 4k71.pdb as input. Interacting residues were identified using the InterfaceResidues.py script in PyMol from pymolwiki.org, and the script reports interaction surface area in Å^2^. DIII of HSA was defined starting at residue 381.

### Statistical analysis

Microsoft Excel for Mac (version 16.35) and GraphPad Prism 8 for Mac (Version 8; GraphPad Software Inc.) were used for data analysis, while GraphPad Prism 8 was used for two-tailed statistical analysis with unpaired t test, multiple comparison two-way ANOVA (95% CI, *P* < 0.05 considered statistically significant) and to calculate EC_50_ values. Figures were made with GraphPad Prism, Adobe Illustrator, and BioRender.com.

## Supplementary Material

pgaf042_Supplementary_Data

## Data Availability

Source data are included in Supplementary material.
