# Supplementary material for: Engineering of anticancer human immunoglobulin A equipped with albumin for enhanced plasma half-life
Source: PNAS Nexus. 2025 Feb 11;4(2):pgaf042. doi: 10.1093/pnasnexus/pgaf042 (PMC11878800; doi:10.1093/pnasnexus/pgaf042)
Supplement: pgaf042_Supplementary_Data [file pgaf042_supplementary_data.zip › PNASNEXUS_supplementary.pdf]

PNAS nexus

Supporting Information for

Engineering of anti-cancer human IgA equipped with albumin for enhanced plasma half-life

Simone Mester, Chilam Chan, Marta Lustig, Stian Foss, J. H. Marco Jansen, Marie Leangen Herigstad, Mitchell Evers, Jeannette Nilsen, Karli R. Reiding, J. Mirjam A. Damen, Renate Burger, Algirdas Grevys, Bjørn Dalhus, Thomas Valerius, Inger Sandlie, Jeanette H.W. Leusen and Jan Terje Andersen

Paste corresponding author name: Jan Terje Andersen
Email: j.t.andersen@medisin.uio.no

This PDF file includes:

Supporting text
Figures S1 to S9
Tables S1 to S2

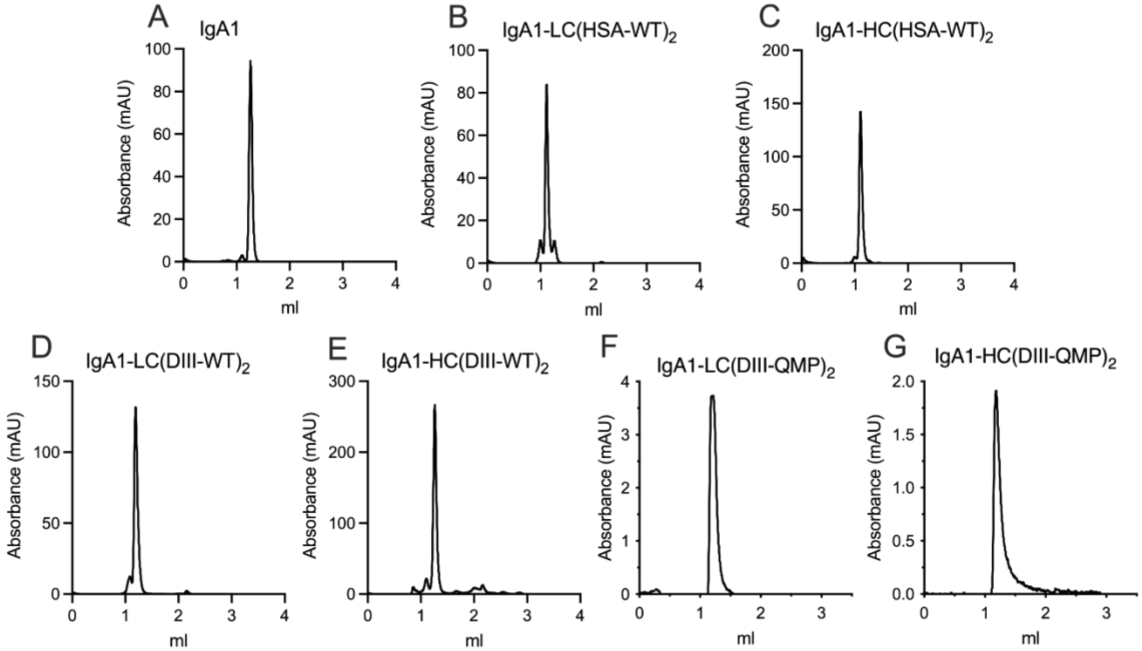

Fig. S1: Isolation of monomeric IgA1 variants by size exclusion chromatography. A-G) Elution profiles of anti-HER2 IgA1, IgA1-HSA, IgA1-DIII-WT and IgA1-DIII-QMP from analytical size exclusion chromatography. All IgA variants appeared as monomeric peaks corresponding to expected molecular masses

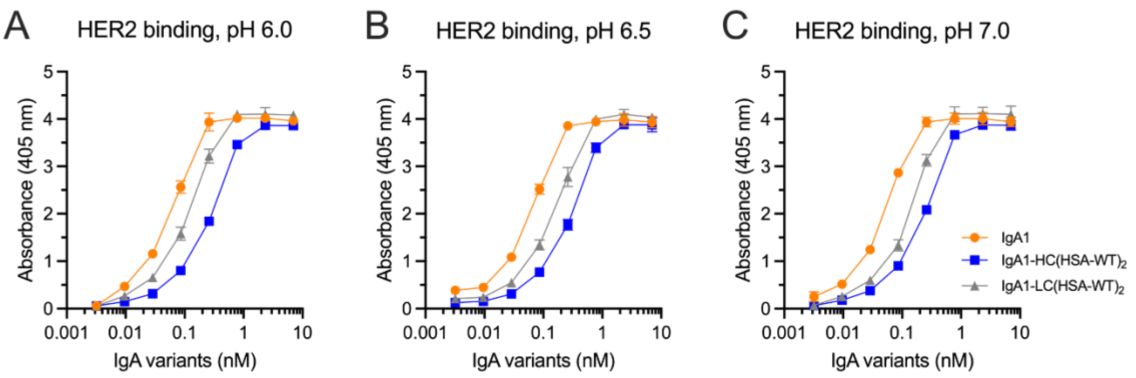

Fig. S2. HSA-fused IgA1 binding to HER2 through a pH gradient. (A-C) ELISA results showing binding of titrated amounts of IgA1 and IgA1-HSA fusions to recombinant HER2 coated in wells at (A) pH 6.0, (B) pH 6.5 (C) pH 7.0. Shown as mean ± SD of duplicates.

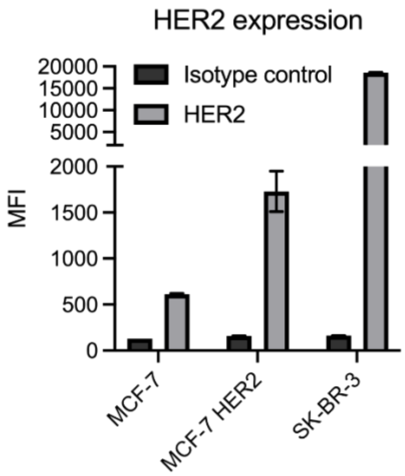

Fig. S3: HER2 expression on MCF-7, MCF-7-HER2 and SK-BR-3 cell lines. The HER2 expression levels on MCF-7, MCF-7 HER2 and SK-BR-3 cell was assessed using an anti-human HER2 mouse IgG1 antibody, followed by F(ab')2 Fragment of FITC-Conjugated Goat Anti-Mouse Immunoglobulins to detect HER2 expression by flow cytometry. Isotype control was used as negative control.

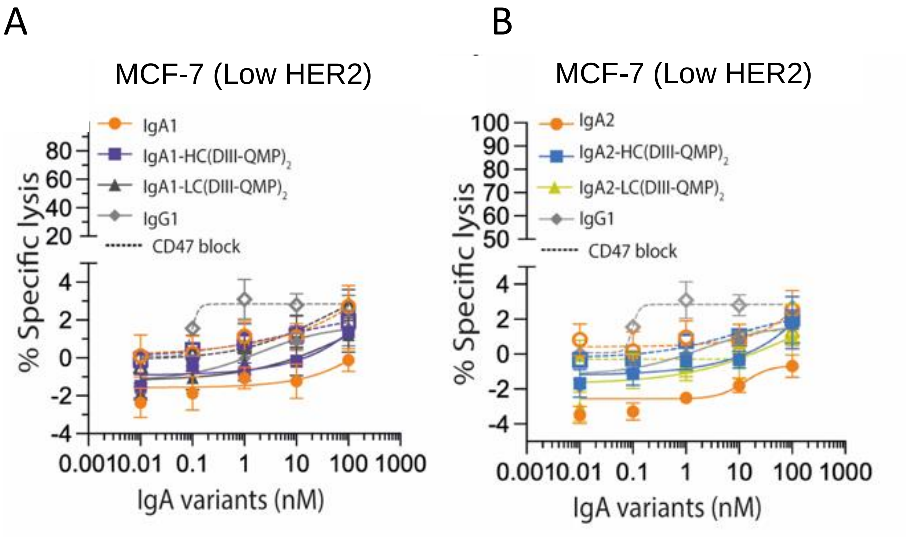

Fig. S4. ADCC activity of DIII-QMP fused IgA1 at low expression levels of HER2. ADCC results showing the cytotoxic potential of (**A**) IgA1 compared with that of the IgA1-DIII-QMP fusions and (**B**) IgA2 compared with that of the IgA2-DIII-QMP fusions in the presence of PMNs (Effector cell : Target cell (E:T) = 40:1), and in the presence (dotted line) and absence (solid line) of a CD47 blockade using a SIRPα IgG1 PGLALA fusion protein. Shown as mean±SEM of triplicates from three independent experiments with PMNs from different donors. Specific lysis (%) of MCF-7 cells was determined.

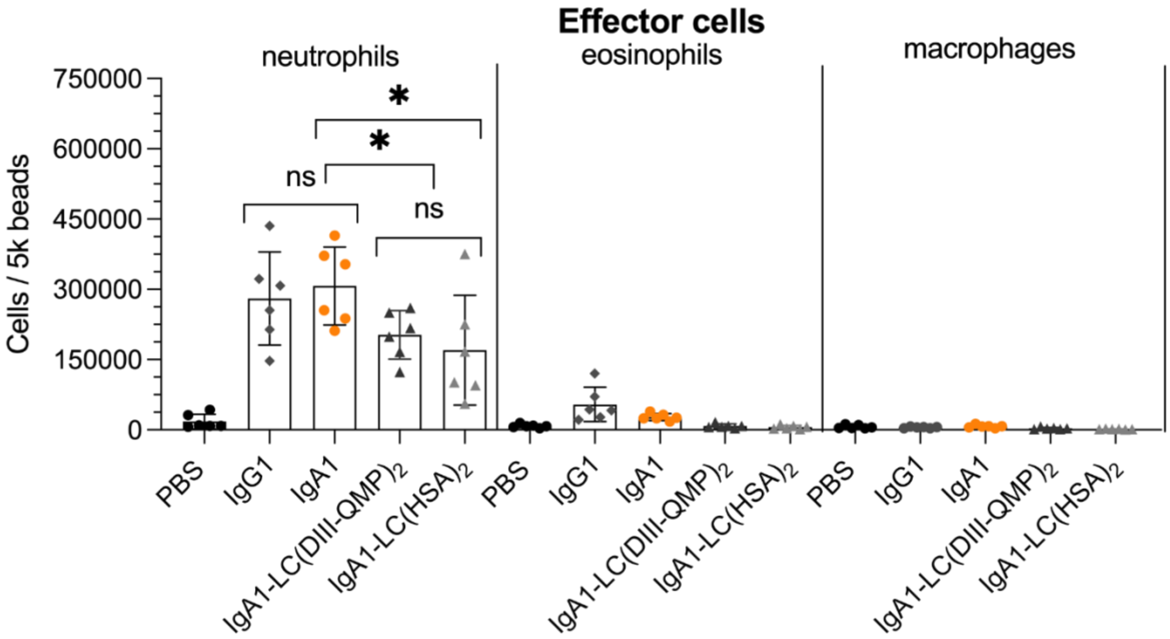

Fig. S5. Effector cells present in the Ba/F3 peritoneal tumor mouse model after treatment. Number of neutrophils, eosinophils and macrophages counted per 5000 beads in mice given PBS anti-Her2 IgG1, IgA1, and the IgA1 DIII-QMP fusion variants n=6 mice per group. Shown as mean ±SD. ns > 0.05, * = 0.0259, 0.0413, by two-tailed analysis using unpaired T-test.

A

B

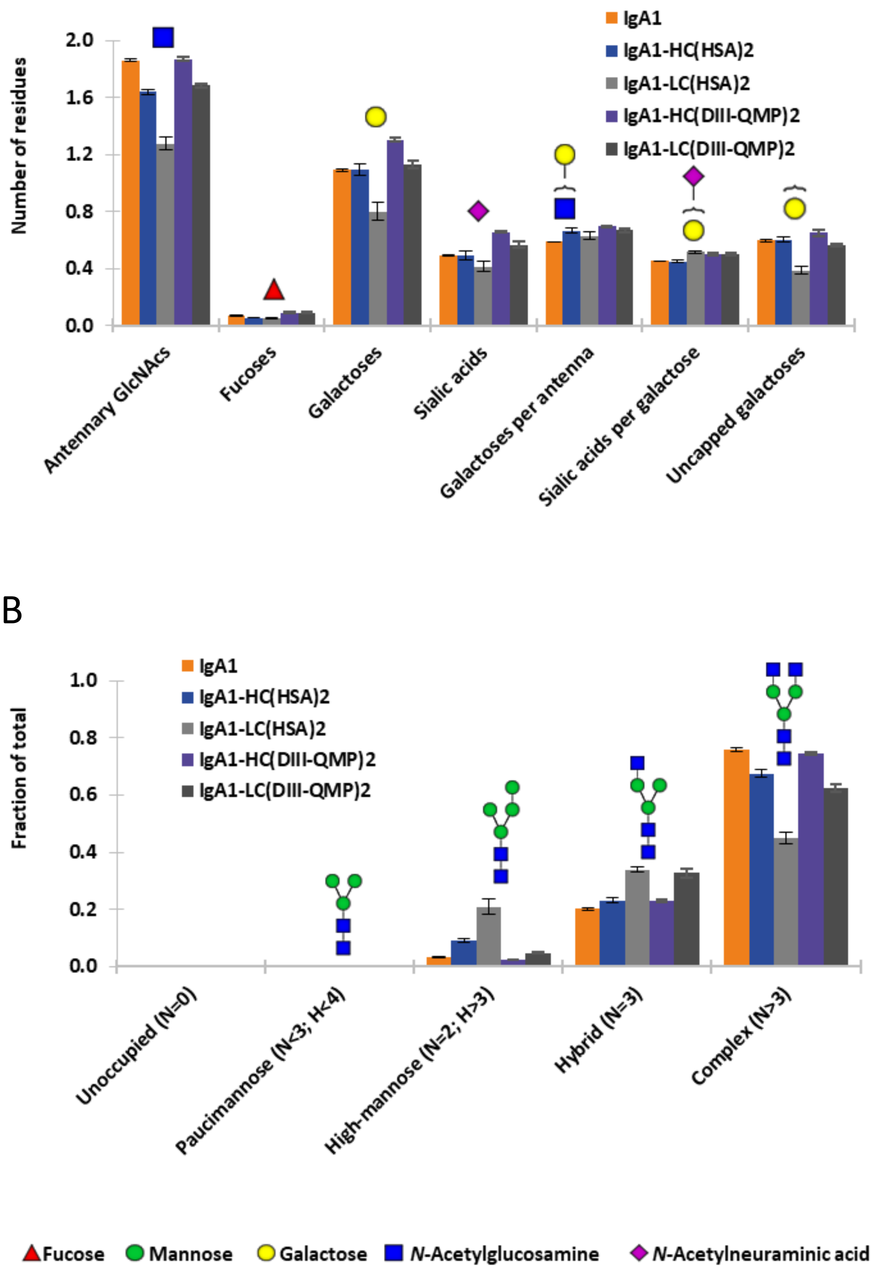

Fig. S6. Glycosylation characteristics of parental IgA1 and IgA1 variants fused to either DIII or full-length HSA mapped by MS. A-B) The data are presented as the average MS1 areas ± SD of the technical triplicates.

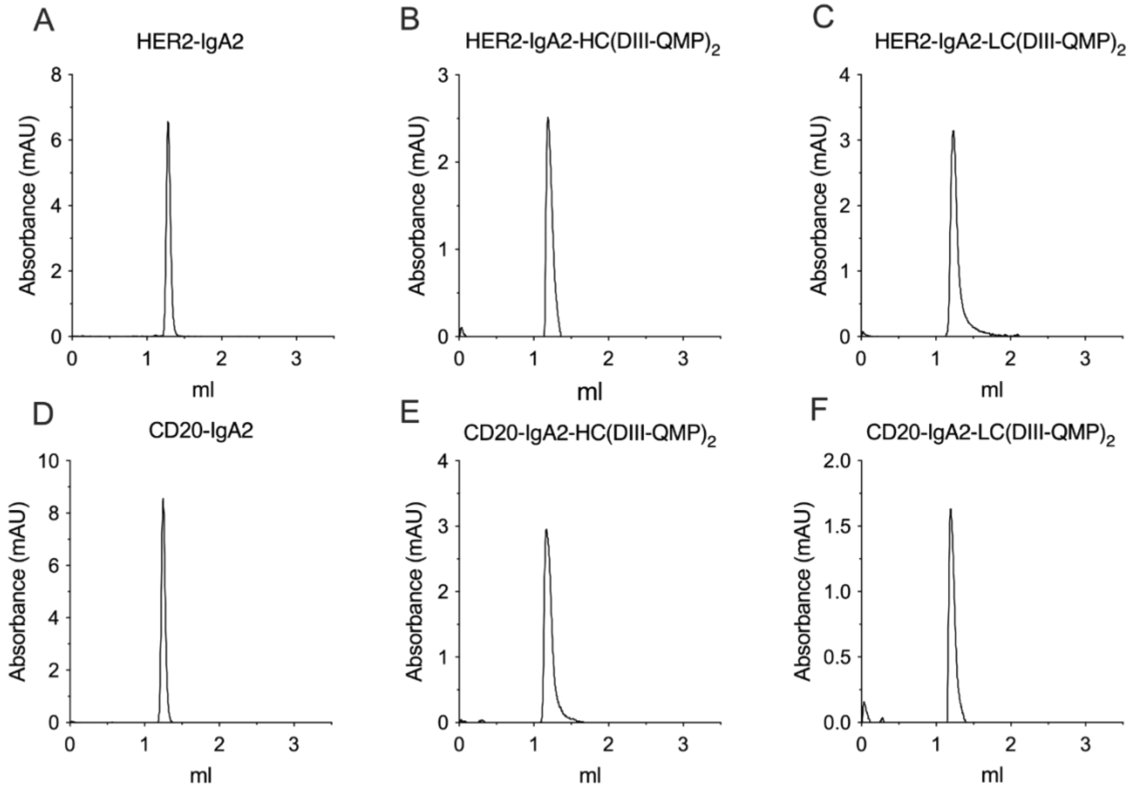

Fig. S7: Isolation of monomeric IgA2 variants by size exclusion chromatography. A-F) Elution profiles of the HER2 and CD20 IgA2 and IgA2-DIII-QMP fusions from analytical size exclusion chromatography. All IgA2 variants appeared as monomeric peaks corresponding to expected molecular size.

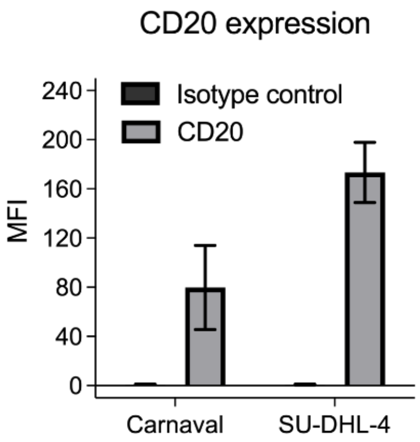

Fig. S8. CD20 expression on Carnaval and SU-DHL-4 cell lines. An anti-human CD20 mouse IgG2 antibody was used together with FITC-conjugated goat anti-mouse IgG Fc-specific F(ab)$_2$ fragments to detect CD20 expression by flow cytometry on Carnaval and SU-DHL-4 B-cell lines. Isotype control was used as negative control used as negative control.

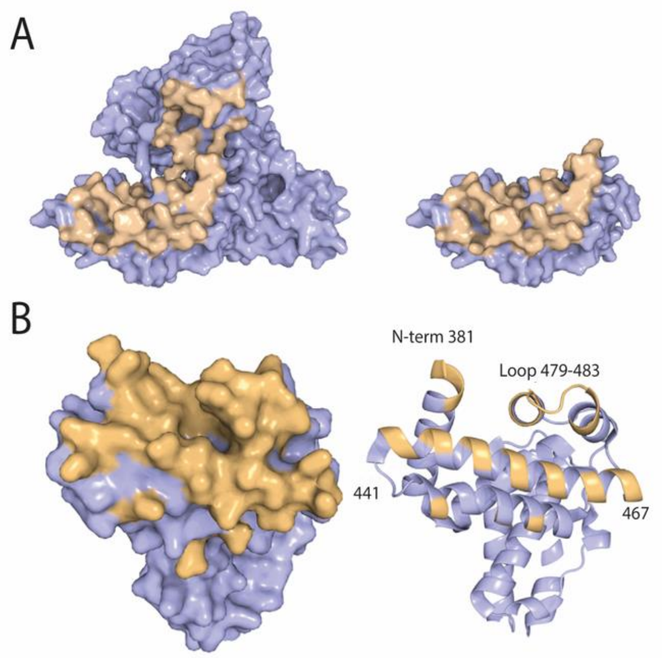

Fig. S9. Interaction surfaces of HSA and DIII. (**A**) The human FcRn interaction surface is shown in yellow, while the rest of full-length human albumin (1815Å^2) (left) and DIII (1373Å^2) (right) shown in purple. (**B**) Surfaces exposed DIII areas shown in yellow in the absence of DI and DII of human albumin. The surfaces include the N-terminal end of DIII starting at residue 381, residues along the alpha-helix (441-467) as well as loop corresponding to residues 479-483. The interaction surface analysis was performed in PyMOL using the human FcRn-HSA complex 4k71.pdb as input.

Table S1. Calculated EC50 from HER2 binding ELISA.		
HER2 IgA variant	EC50 (nM)	Reference to Fig.
IgA1	0.05815	Fig. 1C (pH 7.4)
IgA1-HC(HSA-WT)$_2$	0.2741	Fig. 1C (pH 7.4)
IgA1-LC(HSA-WT)$_2$	0.1210	Fig. 1C (pH 7.4)
IgA1	0.06729	Fig. S2A (pH 6.0)
IgA1-HC(HSA-WT)$_2$	0.2961	Fig. S2A (pH 6.0)
IgA1-LC(HSA-WT)$_2$	0.1615	Fig. S2A (pH 6.0)
IgA1	0.05304	Fig. S2B (pH 6.5)
IgA1-HC(HSA-WT)$_2$	0.2340	Fig. S2B (pH 6.5)
IgA1-LC(HSA-WT)$_2$	0.1411	Fig. S2B (pH 6.5)
IgA1	0.07826	Fig. S2C (pH 7.0)
IgA1-HC(HSA-WT)$_2$	0.4810	Fig. S2C (pH 7.0)
IgA1-LC(HSA-WT)$_2$	0.2659	Fig. S2C (pH 7.0)
IgA1	0.5388	Fig. 3B (pH 7.4)
IgA1-HC(DIII-WT)$_2$	0.7903	Fig. 3B (pH 7.4)
IgA1-LC(DIII-WT)$_2$	0.9712	Fig. 3B (pH 7.4)
IgA1	0.2078	Fig. 4B (pH 7.4)
IgA1-HC(DIII-QMP)$_2$	0.2005	Fig. 4B (pH 7.4)
IgA1-LC(DIII-QMP)$_2$	0.2508	Fig. 4B (pH 7.4)
IgA2	0.2455	Fig. 6B (pH 7.4)
IgA2-HC(DIII-QMP)$_2$	0.1914	Fig. 6B (pH 7.4)
IgA2-LC(DIII-QMP)$_2$	0.1703	Fig. 6B (pH 7.4)

Table S2. Non-compartmental PK analysis (NCA). NCA PK model parameters were determined from the measured antibody concentrations in plasma using gPKPDsim for MatLab.

HER2 IgA variant	Dose (mg/kg)	Route	AUC (μg*d/mL)	Cmax (μg/mL)	CL (mL/d/kg)	MRT (d)	Vss (mL/kg)	$T_{1/2}$ (days)
IgA1	1.0	IV Bolus	9.6	3.2	105.3	1.3	135.9	0.9
IgA1-HC(HSA-WT)$_2$	1.9	IV Bolus	512.4	60.4	1.5	13.3	20.9	10.0
IgA1-LC(HSA-WT)$_2$	1.9	IV Bolus	518.9	60.8	1.93	14.0	20.1	9.61
IgA1-HC(DIII-WT)$_2$	1.3	IV Bolus	33.5	10.5	32.9	1.1	37.0	0.9
IgA1-LC(DIII-WT)$_2$	1.3	IV Bolus	15.2	4.8	66.5	1.3	87.9	1.0
IgA1-HC(DIII-QMP)$_2$	1.3	IV Bolus	63.0	12.4	16.0	3.3	53.7	3.1
IgA1-LC(DIII-QMP)$_2$	1.3	IV Bolus	70.3	14.5	11.9	6.5	78.0	4.1
IgA2	1.0	IV Bolus	3.1	1.0	328.5	1.2	403.7	0.7
IgA2-HC(DIII-QMP)$_2$	1.3	IV Bolus	100.0	18.8	10.0	3.7	37.5	5.1
IgA2-LC(DIII-QMP)$_2$	1.3	IV Bolus	45.0	11.3	22.3	2.5	58.1	2.2

AUC = Area under the curve from time 0 to the last measured time point.

C_{max} = Maximum concentration following administration.

CL = Clearance.

MRT = Mean residency time.

Vss = Volume of distribution at steady state.

$T_{1/2}$ = Terminal plasma half-life.
